# BreastNet18: A High Accuracy Fine-Tuned VGG16 Model Evaluated Using Ablation Study for Diagnosing Breast Cancer from Enhanced Mammography Images

**DOI:** 10.3390/biology10121347

**Published:** 2021-12-17

**Authors:** Sidratul Montaha, Sami Azam, Abul Kalam Muhammad Rakibul Haque Rafid, Pronab Ghosh, Md. Zahid Hasan, Mirjam Jonkman, Friso De Boer

**Affiliations:** 1 Department of Computer Science and Engineering, Daffodil International University, Dhaka 1207, Bangladesh; sidratul15-11685@diu.edu.bd (S.M.); rakibul15-11463@diu.edu.bd (A.K.M.R.H.R.); zahid.cse@diu.edu.bd (M.Z.H.); 2 College of Engineering, IT and Environment, Charles Darwin University, Darwin, NT 0909, Australia; mirjam.jonkman@cdu.edu.au (M.J.); friso.deboer@cdu.edu.au (F.D.B.); 3 Department of Computer Science (CS), Lakehead University, 955 Oliver Rd, Thunder Bay, ON P7B 5E1, Canada; pghosh1@lakeheadu.ca

**Keywords:** mammograms, image preprocessing, fine-tuned VGG16, deep learning, breast cancer classification, data augmentation, ablation study, transfer learning models, feature map analysis, CBIS-DDSM

## Abstract

**Simple Summary:**

Breast cancer diagnosis at an early stage using mammography is important, as it assists clinical specialists in treatment planning to increase survival rates. The aim of this study is to construct an effective method to classify breast images into four classes with a low error rate. Initially, unwanted regions of mammograms are removed, the quality is enhanced, and the cancerous lesions are highlighted with different artifacts removal, noise reduction, and enhancement techniques. The number of mammograms is increased using seven augmentation techniques to deal with over-fitting and under-fitting problems. Afterwards, six fine-tuned convolution neural networks (CNNs), originally developed for other purposes, are evaluated, and VGG16 yielded the highest performance. We propose a BreastNet18 model based on the fine-tuned VGG16, changing different hyper parameters and layer structures after experimentation with our dataset. Performing an ablation study on the proposed model and selecting suitable parameter values for preprocessing algorithms increases the accuracy of our model to 98.02%, outperforming some existing state-of-the-art approaches. To analyze the performance, several performance metrics are generated and evaluated for every model and for BreastNet18. Results suggest that accuracy improvement can be obtained through image pre-processing techniques, augmentation, and ablation study. To investigate possible overfitting issues, a k-fold cross validation is carried out. To assert the robustness of the network, the model is tested on a dataset containing noisy mammograms. This may help medical specialists in efficient and accurate diagnosis and early treatment planning.

**Abstract:**

Background: Identification and treatment of breast cancer at an early stage can reduce mortality. Currently, mammography is the most widely used effective imaging technique in breast cancer detection. However, an erroneous mammogram based interpretation may result in false diagnosis rate, as distinguishing cancerous masses from adjacent tissue is often complex and error-prone. Methods: Six pre-trained and fine-tuned deep CNN architectures: VGG16, VGG19, MobileNetV2, ResNet50, DenseNet201, and InceptionV3 are evaluated to determine which model yields the best performance. We propose a BreastNet18 model using VGG16 as foundational base, since VGG16 performs with the highest accuracy. An ablation study is performed on BreastNet18, to evaluate its robustness and achieve the highest possible accuracy. Various image processing techniques with suitable parameter values are employed to remove artefacts and increase the image quality. A total dataset of 1442 preprocessed mammograms was augmented using seven augmentation techniques, resulting in a dataset of 11,536 images. To investigate possible overfitting issues, a k-fold cross validation is carried out. The model was then tested on noisy mammograms to evaluate its robustness. Results were compared with previous studies. Results: Proposed BreastNet18 model performed best with a training accuracy of 96.72%, a validating accuracy of 97.91%, and a test accuracy of 98.02%. In contrast to this, VGGNet19 yielded test accuracy of 96.24%, MobileNetV2 77.84%, ResNet50 79.98%, DenseNet201 86.92%, and InceptionV3 76.87%. Conclusions: Our proposed approach based on image processing, transfer learning, fine-tuning, and ablation study has demonstrated a high correct breast cancer classification while dealing with a limited number of complex medical images.

## 1. Introduction

Breast cancer is one of the leading causes of death for women worldwide [1] and is occurring more frequently in both developed and developing countries. In 2012 the number of new cases of breast cancer was about 1.6 million [2]. In the year of 2020 the number of new cases for breast cancer had risen to 2.2 million, 11.7% [3] of all new cancer cases that year. Among all cancer patients 684,996 [3] died from breast cancer in 2020. According to the World Health Organization (WHO), the number of expected cancer cases in 2025 will be approximately 19.3 million [4]. However, accurate tumor identification and distinguishing malignant from benign tumors at an early stage crucially impacts on chances of survival [5]. Early detection greatly improves the chances of survival [6]. A recent study found that 5 year survival rates increases to 91% with early diagnosis [7]. This is compounded by the fact that there is a lack of radiologists and doctors across the world who can interpret the screening results, especially in regional areas and developing countries [8]. As time is a major factor in saving lives in the case of breast cancer, human resources and technology to deliver prompt patient services in terms of screening, diagnosis, and treatment are essential [9]. However, tumor diagnosis is time-consuming and often challenging for radiologists, by examining medical images due to the presence of noise, artefacts, and complex structure. This is compounded by the fact that there is a lack of radiologists and doctors across the world who can interpret the screening results, especially in regional areas and developing countries [8]. Additionally, a growing number of patients adds radiologist’s burden that often results in misdiagnosis of tumors. At present, mammography, magnetic resonance imaging (MRI), and ultrasound are the most common medical imaging modalities, available and used for early detection of cancerous breast tumors. In this regard, mammogram based diagnosis [10] outperforms symptoms based diagnosis among other modalities. Digital mammography provides high-quality images at a relatively low radiation dose, with a wide dynamic range and low noise [11]. To detect the cancerous lesion, radiologists examine the region, size, shape, density of masses, and calcifications as well as distortions presented on a mammogram [12]. Distinguishing a mass from adjacent dense tissue is often extremely challenging, even for experts. Moreover, in mammographic studies, detecting the chest wall is often challenging due to deficiency of regularity, low perceptibility of the chest wall, and large heterogeneity of breast tissue [13]. The pectoral muscle and the chest wall has nearly the equivalent density as the dense tissues of the breast which can cause poor performance in tumor detection [14]. In MRI of dense breasts, it is observed that the visual appearance of fibroglandular tissue and the chest wall look relatively similar. As a consequence, the delineation and exclusion of the muscle while preserving the necessary dense tissue is challenging [15].

The accessible mammogram datasets are extremely imbalanced, comprising of an inadequate number of images. Image processing techniques along with machine learning could be a valuable tool for detection and diagnosis but also often results in false positive and false negative cases [6]. Deep Learning technology can detect breast cancer in an early stage by decreasing mammogram interpretation time [16]. Currently, a host of Deep Learning algorithms are available but not all have been explored for their usefulness in detecting breast cancer. These algorithms are used for fully automated mass segmentation, detection, and classification by extracting important distinct features from images without manual human interference. When the number of images is insufficient to train a Deep CNN (DCNN) network, transfer learning (TL) plays a vital role in boosting diagnosis performance in the medical domain, especially when dealing with the complex characteristics of breast mammograms. Recently, fine-tuned TL networks with pre-trained weights are gaining interest of scholars by solving complex classification tasks with significant interpretation performance.

**Figure 1:**
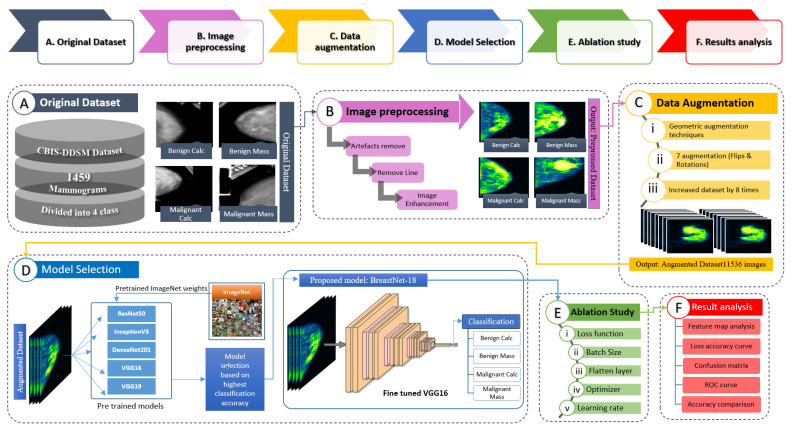
Working flow of the entire classification process where each step is represented by a block. Block (**A**) represents the original mammogram dataset from CBIS DDSM. (**B**) Various image preprocessing steps applied to the original dataset result in a preprocessed dataset. (**C**) Data augmentation techniques applied to the preprocessed dataset to increase volume of the dataset, resulting in an augmented dataset, (**D**) experimenting with various pre-trained models and selecting the model based on highest classification accuracy on the augmented dataset, results in the proposed BreastNet18 model (fine-tuned VGG16). (**E**) An ablation study conducted on proposed model results in a more robust network with higher accuracies and (**F**) results of the final model are analyzed with performance matrices along with feature map analysis.

The aim of this study is to detect and classify breast cancer into benign calcifications (BC), benign masses (BM), malignant calcifications (MC), and malignant masses (MM) at initial stage in order to reduce the risk of death by assisting the experts in better and proper treatment planning. All the experiments of this study are conducted using the Curated Breast Imaging Subset of the Digital Database for Screening Mammography (CBIS-DDSM) dataset. Mammograms of the CBIS-DDSM dataset contain noise and artefacts, lines on the border of the image and lines attached to breast portion of the image. Removal of artefacts is crucial in order to achieve good performance from a CNN model. Moreover, resemblance between dense breast tissue and tumor regions might lead to poor interpretation capability of the model. To address this issue, the tumorous lesion can be enhanced by properly balancing brightness and contrast levels of the raw mammograms. A fully automated and reliable deep learning model namely BreastNet18 based on transfer learning and ablation study is proposed in this research, to classify cancerous lesions on breast mammograms. The entire process of mammography classification proposed in this research is illustrated in Figure 1. To develop the model, the performance of six existing pre-trained and fine-tuned TL architectures namely VGG16, VGG19, MobileNetV2, ResNet50, DenseNet201, and InceptionV3 are compared to determine which model records the highest accuracy. Each of the models is fine-tuned by freezing the pre-trained weights of the primary layers and retraining the last layer using our breast mammogram dataset. An ablation study is performed to the model which yields the highest accuracy (VGG16) using four study cases to further increase the overall model’s accuracy and robustness. Performance matrices are investigated to evaluate the robustness of our model. Additionally, a number of image pre-processing and augmentation methods are investigated to achieve better performance. The preprocessing algorithms and their parameter values are chosen through a vast experiment on our dataset. Statistical analysis including Peak signal-to-noise ratio (PSNR), Mean Squared Error (MSE) and Root Mean Squared Error (RMSE), structural similarity index measure (SSIM), and histogram analysis on the preprocessed images is done to evaluate these image processing algorithms. For the proposed model, feature maps are generated for every CNN block to show how the detailed information is extracted. Once the proposed model yields optimal performance, a k-fold cross validation is carried out to ensure there is no occurrence of overfitting. Moreover, to check whether the model can sustain its performance for a real world dataset, it is tested on a noise induced dataset. The consistent outcomes of this research demonstrate that image processing techniques and ablation study integrated with transfer learning method can accelerate the diagnosis which could greatly assist clinical experts in the treatment of breast cancer.

## 2. Research Aim and Scope

The aim of this study is to construct an effective automated deep learning method to assist radiologists by classifying cancerous lesions into benign calcifications, benign masses, malignant calcifications, and malignant masses with accurate and fast performance. The objectives and approach can be summarized as follows:

- Initially, artefacts and noise presented in raw mammograms are removed by applying noise reduction and data pre-processing algorithms, the quality of the mammograms is improved and the breast lesion emphasized with different data enhancement methods.
- Preprocessing algorithms and their parameter values which result in the best outcome, after experimentation with our dataset, are selected.
- Statistical analysis such as MSE, PSNR, SSIM, RMSE, and histogram analysis is performed on the pre-processed images to ensure the quality and pixel information has not deteriorated.
- The number of mammograms is increased eight times using seven data augmentation techniques to deal with overfitting issues and the dataset is split into train, validation, and test sets.
- Pre-trained networks namely VGGNet16, VGGNet19, ResNet50, InceptionV3, MobileNetV2, and DenseNet201 are modified by fine-tuning the model’s final layers to determine the best classifier for the augmented dataset.
- BreastNet18 model is proposed based on a fine-tuned VGG16 architecture after thorough experimentation with our dataset as it yields the highest accuracy among the pre-trained models.
- An ablation study is conducted on the proposed model in order to improve its classification performance.
- To avoid the possibility of overfitting, the model is evaluated by employing k-fold cross validation technique.
- Once the model offers adequate performance, to further investigate the robustness, it is tested on a noise induced dataset.

## 3. Literature Review

Early detection of breast cancer and classification of mammogram images with the help of different Deep Learning Classifiers is a major area of research. However, there is a limited availability of labeled mammographic images which affects the viability of training a CNN from scratch [17]. In a recent study [18], it was found that a pre-trained model provides a better performance on a DDSM dataset and is able to minimize the loss function during the gradient descent process. This was implemented using various image classification models (AlexNet, VGG16, VGG19, MVGG, Mobile Net, and ResNet50) which were modified by tuning the feed-forward neural networks. The proposed hybrid model, a combination of MVGG and ImageNet, performed best and provided an accuracy of 94.3%, a precision of 93.5%, a sensitivity of 93.7% and an F1-score of 93.7%. Two experiments were conducted, to classify cancerous and non-cancerous mammography lesions. The authors introduced five transfer learning models in the first approach while in the second approach; a support vector machine was applied. They also employed several image processing and augmentation techniques to improve the classification performance but did not describe the processes in detail in their paper. Recorded results showed that, their proposed ConvNet + SVM model attained a training accuracy of 97.7% with validating accuracy of 97.8%. Furthermore, pre-trained and fine-tuned VGGNet16 model acquired accuracy of 90.2%, VGGNet 93.5%, GoogLeNet 63.4%, MobileNetV2 82.9%, ResNet50 75.1%, and DenseNet121 72.9% [19]. Other authors [20] combined various convolution and capsule features in a feature fusion method to derive an updated feature set. These new features are more notable than convolution or capsule features and have a better classification performance. Their network was able to simultaneously extract capsule features and convolution features and achieved an accuracy of 94.52%. Mammographic mass detection is one of the most important areas of Computer Aided Diagnosis (CAD) and can be achieved by using DCNN as a feature extractor [21]. These authors combined scoring features, Gray-Level Co-occurrence Matrix (GLCM) and Histogram of Oriented Gradient (HOT) as input features. Support Vector Machine (SVM) and Extreme Gradient Boosting (XGBoost) were trained using these inputs. They found that an XGBoost classifier performed better than SVM, obtaining an accuracy of 84%. Hamid et al. [22] proposed an architecture based on VGG16 and VGG19 models for the automatic classification of breast cancer from histopathology images. This hybrid classifier resulted in an accuracy of 95.29%, 97.73% sensitivity, and an F1 score of 95.29%. Other authors [23] evaluated CNN to classify mammograms in three stages: abnormal or normal, mass or calcification and finally malignant or benign. In classifying normal and abnormal mammograms, VGG19 obtained the best result with an AUC of 94% and an accuracy of 93.45%. The second-best result was obtained with their proposed Multi-view Features Fusion (MVFF) model with 93.4% AUC and 93.73% accuracy. In the classification of abnormality type, an accuracy of 92.29% was achieved in the third stage, their MVFF model achieved an accuracy of 77.66%. Wang et al. [24] proposed a new method based on the combination of deep features with CNN. However, they only used 400 mammogram images, with 200 images of malignant masses and 200 of benign masses. Extreme learning machine (ELM) and support vector machine (SVM) classifiers were used for classification. ELM outperformed the SVM classifier with an accuracy of 86.50%. Li et al. [5], proposed an improved neural network model called DenseNet-II for the classification of benign and malignant mammography images. In their study, five different models were compared. They recorded an accuracy of 94.55% using a DenseNet-II classifier. VGG16, VGG19, and ResNet50 are pre-trained networks known because of their wide and deep architectures. VGG16 and VGG19 hold the first position for localization and the second position for classification in the ImageNet Large Scale Visual Recognition Challenge (ILSVRC-2014) [25]. In this experiment, three pre-trained networks (VGG16, VGG19, and ResNet50) were compared and found that a hybrid approach performed better than a fully trained network, obtaining an accuracy of 92.60%, an area under curve (AUC) of 95.65%, and a precision score of 95.95%. Ribli et al. [26] proposed a CAD system based on a successful object detection framework called Faster R-CNN. They used VGG16 network as the base of CNN achieving a 95% AUC score for classification. Another new CAD system for the classification of breast masses into benign and malignant tumors was developed by Ragab et al. [4] using two different segmentation approaches. Similarly, a DCNN model was utilized [21] for the extraction of classification features. A fine-tuned AlexNet with SVM resulted in an accuracy of 87.2%. CNN performs well on a large number of datasets [27] due to its self–learning strategies. An AlexNet-like architecture had an AUC score of around 85% but deeper networks and with an increased number of kernels resulted in an accuracy of 89.6%. An accuracy of 87.5% was obtained without data augmentation techniques and an accuracy of 92.9% with data augmentation. A completely integrated CAD system using a deep learning classifier to perform detection, segmentation, and classification of mammograms was proposed by Al-antari et al. [28]. For performing segmentation, a full resolution convolutional network (FrCN) was utilized. To detect and classify breast tumor, CNN was also used. The suggested system’s first two stages obtained 98.96% accuracy in mass detection and a 92.97% accuracy in mass segmentation. These segmented mass images were used to classify breast tumors and an overall accuracy of 95.64% was obtained by CNN in this classification stage. A deep feature based framework for breast lesion classification was designed by Jiao et al. [29]. It mainly consisted of a CNN with a series of decision mechanisms. The fine-tuned CNN was trained on a large number of natural images. The proposed model had an improved accuracy of 96.7%. Currently, deep learning models are widely used on image classification applications as they yield good performance with minimal misclassification rate. However, Wang et al. [30] examined whether these models were able to sustain high performance on unseen external data using mammograms. Six deep learning networks were assessed on four mammogram data sets. Digital Database for Screening Mammography or a joint dataset that contained Digital Database for Screening Mammography were employed for training and validation. Three unseen dataset were used to test the models. Though the models were able to attain a good performance on the validation dataset, the performance dropped significantly while testing on external dataset, recording area under the receiver operating characteristic curve scores between 0.44 and 0.65. Paquin et al. [31] developed a CNN model using mammograms on a simplified feature learning and fine-tuned network to distinct cancer from normal mammograms. Their proposed architecture performed best on the DDSM mammogram dataset by attaining accuracy, sensitivity, specificity, and precision of 92.84%, 95.30%, and 96.72% respectively. Data preprocessing, augmentation, and k-fold cross validation was carried out to increase the robustness of model. Jadoon et al. [32] proposed two methods, namely, CNN-discrete wavelet (CNN-DW) and CNN-curvelet transform (CNN-CT) to classify mammograms into normal, benign and malignant cases. Data augmentation was carried out by mammogram patches and contrast limited adaptive histogram equalization(CLAHE) was employed to enhance the mammograms. In the first approach, CNN-DW, the mammograms were decomposed into four subbands in coexistence with two-dimensional discrete wavelet transform (2D-DWT). In the second approach, a discrete curvelet transform (DCT) was employed. Their proposed model resulted in an accuracy of 81.83% and 83.74%, for the first and the second approach, respectively. To classify normal and abnormal mammograms, Cardezi et al. [33] introduced a VGG-16 deep learning model with the Image retrieval in Medical Applications (IRMA) dataset. The model was evaluated employing 10 fold cross validation on SVM, simple logistics, binary trees, and K nearest neighbor (KNN) classifiers with k value of 1, 3, and 5. They achieved 100% accuracy with SVM, KNN, and binary trees. For the simple logistic classifier, an accuracy of 99.9% was achieved while all of the classifiers recorded an AUC value of 1.0. A novel method to detect abnormal lesions in mammograms, combining scale invariant feature transform (SIFT) keypoints and transfer learning with pre-trained CNN models (PyramidNet and AlexNet) was proposed by Bruno et al. [34]. Two datasets were used, both of which were augmented and image patches were extracted. One of these datasets was the public available dataset Mini-MIAS, the other was their own dataset Suspicious Region Detection on Mammogram from PP (SuReMaPP). For mini-MIAS their proposed approach was able to attain a sensitivity of 98% and a specificity of 90%. For SuReMaPP the sensitivity was 94% and the specificity 91%.

## 4. Dataset

A total of 1459 mammograms, provided by CBIS-DDSM, are analyzed for this research. The DDSM is a database of mammograms and the CBIS-DDSM [35] dataset is an updated version of DDSM. CBIS-DDSM contains 398 images with benign calcifications, 417 images with benign masses, 300 images with malignant calcifications with the remaining ones malignant masses. In this dataset, all images are 224 × 224 pixels and in RGB format. The description of the dataset is summarized in Table 1.

**Figure 2:**
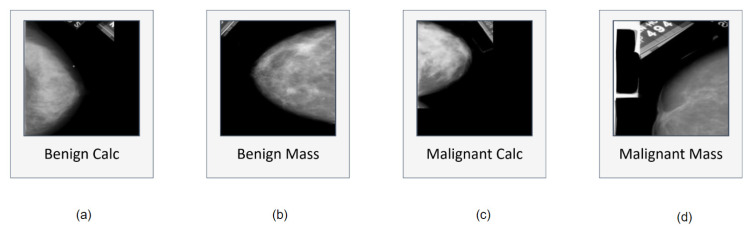
CBIS-DDSM dataset containing mammograms with four classes where various artefacts are present in all classes. Image (**a**) shows small label at the corner of the image, image (**b**) has a large label, image (**c**) contains vertical and horizontal lines attached to the breast part of the image, and image (**d**) contains horizontal line at bottom border of the image.

After getting the dataset, we found some blank images as well as some images with just artefacts classed as malignant masses. These images may cause a reduction of accuracy. We therefore manually removed 17 images. After removing the images we were left with 327 malignant mass mammograms while the number of mammograms in the other classes remained the same as before. As a result, a dataset of 1442 images is generated containing four different classes. Figure 2 showcases examples of four classes along with their characteristics and artefacts.

## 5. Image Preprocessing Techniques

Image pre-processing is regarded to be the first and foremost task before feeding the images into a neural network to achieve a satisfactory accuracy and decrease the computational time of a model. Without applying pre-processing techniques on mammograms, it is difficult for a neural network model to classify them. Therefore, we first focus on image pre-processing. Beeravolu et al. [9] describes diverse pre-processing techniques which can be implemented without affecting the original image quality. Two other studies [4,24] used well-known methods to enhance the contrast of mammograms.

**Figure 3:**
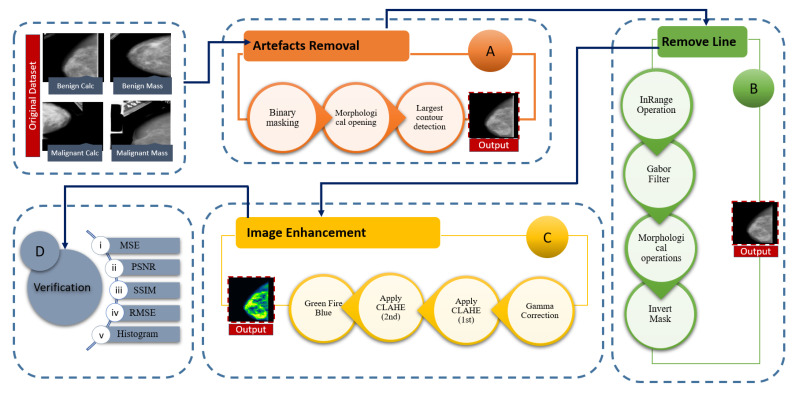
Process diagram of image pre-processing where each step is represented with a block. Block (**A**): artefacts present in the original mammograms are removed using various methods: binary masking, morphological opening, and largest contour detection resulting in artefact free mammograms; (**B**) lines attached to the breast part of the mammograms are removed using various methods: inRange Operation, Gabor filter, Morphological opening, and Invert mask operation; (**C**) image enhancement techniques: Gamma correction, CLAHE, and Green Fire Filter of ImageJ software; and finally in Block (**D**) quality of the enhanced images is verified using image statistical analysis.

In this section, we describe how the quality and quantity of mammograms can be boosted. This includes background removal, artefact removal, and image enhancement. Figure 3 illustrates the main processes and sub-processes of the image pre-processing step. The main processes include artefacts removal, line removal, image enhancement and verification.

Firstly, artefacts are removed from the mammograms followed by some algorithms (binary masking, morphological opening [36] and largest contour detection [37]) to get a more accurate result. Secondly, a “remove line” step is performed to get rid of the vertical line attached with the breast contour. Previously described approaches, such as inRange operation [38], Gabor filter [39], morphological operations [40], and inverse mask methods are used in this research. The sub-process morphological operation has two other sub-processes: opening and dilation. Thirdly, Image enhancement, a procedure of improving the brightness and contrast of original images, is applied to make the cancerous lesion more visible. The sub processes of this step are gamma correction [41], CLAHE(1st) [42], CLAHE(2nd) and a filter of ImageJ software: green fire blue [9]. After applying CLAHE, an improvement of visibility can be noticed. CLAHE is then employed again to improve the contrast level. In other words, CLAHE is applied twice in our process. We did not apply CLAHE a third time as this may affect the details of mammograms by over-amplifying the contrast level. Finally, in the verification section, assessment techniques such as, MSE, PSNR, SSIM, RMSE, and Histogram Analysis are applied to the processed images to evaluate the produced results.

The complete image pre-processing flow is illustrated in Figure 3. The output of a previous step is employed as the input of next step (e.g., the output of binary making is the input of morphological opening). A resulting image after every process is also shown.

### 5.1. Artefacts Removal

Sometimes, undesired objects or areas accidentally appear in images as shown in Figure 2. This may hamper the performance of the model; hence, successful background removal is a crucial step in image pre-processing. As illustrated in Figure 2 some images contain artefacts as well as a thin white border vertically or horizontally. In most cases this border appears in the left, right or top edge of the images. We have therefore applied binary masking, morphological opening, and Largest Contour detection to remove these artefacts.

#### 5.1.1. Binary Masking

**Figure 4:**
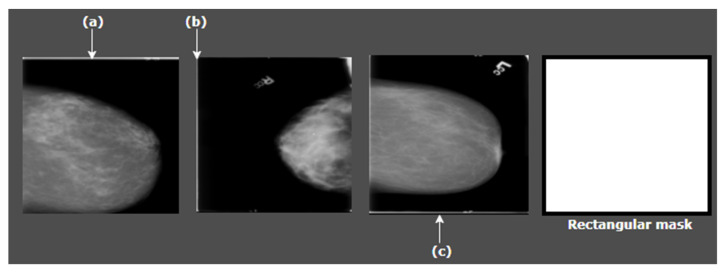
Thin borders of the mammograms, (**a**) border at top, (**b**) border at left, and (**c**) border at bottom.

All kind of images, texts, and videos are stored in computer as a series of binary digits (zeroes or ones). With python we can manipulate every individual bit and decide which to keep and which to remove. In a binary mask, there are two bits where white denotes ‘1’ and black denotes ‘0’. By applying the mask on an image, pixels can be filtered out when the mask [pixel] = 0 and the desired bits can be kept when the mask [pixel] = 1. OpenCV is a tool of python which allows creating binary structural elements of different shapes and sizes. As stated previously, most of the mammograms we are working with contain a thin border either on the top, the bottom, the left or the right side (Figure 4). Removal of this border is crucial as the intensity of tumor and this border are nearly similar which might affect the classification performance. Several approaches such as cropping could be applied for the purpose. However, cropping can result in losing necessary details by shrinking the image. Hence, to get the best outcome, binary masking is applied with a rectangular mask.

The cv2.rectangle () method is used to create a rectangular mask of the same dimensions as our input image. This method requires five parameters, input_image, start_point, end_point, border_color and border_thickness. Input_image is the image to which the mask will be applied, start_point refers to the starting coordinates of the rectangular mask (x and y coordinates), end_point represents the end coordinates of the rectangular mask (x and y coordinates), border_color is the color of the border, and border_thickness is the thickness of the border. As the size of the image border which needs to be removed is not large, the thickness of the binary mask border can be taken as only 5 px so that the border is removed without losing any necessary pixels or information. Therefore, we use a tuple of color (0, 0, 0) as a border_color and a thickness of 5 px as border thickness. After creating the mask, a bitwise_AND is performed to apply the mask on original images. Bitwise AND requires two parameters, the original image and the binary mask. For any bit presented in the input, the output bit will be 0 if the mask bit is 0.

**Figure 5:**
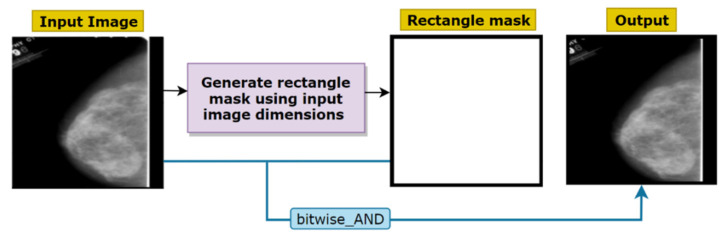
Border removal with binary masking using a rectangle mask.

In this step, after loading the input image, a binary mask is constructed of the same dimension. Finally, the mask and the input are merged. The white edges have been removed successfully from images by applying binary masking which is illustrated in Figure 5.

#### 5.1.2. Morphological Opening

Before applying morphological opening, the image is binarized as our goal is to acquire a binary mask. The cv2.threshold function is used, which is a global thresholding method. This function requires three arguments, input image, threshold value and maximum value. In our case, binary thresholding is applied with a threshold value of 127 and a maximum value of 255. Several other threshold values were tried but 127 produced the best outcome. The pseudo-code for this algorithm is shown below (Algorithm 1):
**Algorithm 1** Binary thresholding**1. START**2.   **READ** source image, s(x,y) where x and y denotes the pixel coordinates3.   threshold value, t = 1274.   maximum value, m = 2555.   **FOR** pixel in s(x,y):6.     **IF** s[pixel] < t:7.       s[pixel] = 0**8.     ELSE**9.       s[pixel] = 255**10.      END IF****11.   END FOR****12. END**

Morphological opening is applied on this mask and after removing artefacts, the mask will be merged with the original image. Morphological operations such as bitwise filtering by a given kernel can produce noteworthy outcomes in terms of removing small noise and irrelevant artefacts from image. Over the last decades, different morphological operations have proven their worth. Morphological opening [40], a function of openCV, is useful in this regard. Morphological opening can be defined as:

(1) X○B=(X⊖B)⊕B

where X is the input image, B is the kernel, X○B is the opening of X by B, X⊖B is the erosion of *X* by *B*, and (X⊖B)⊕B is the dilation of X⊖B by *B*. Morphological opening is the combination of erosion and dilation.

(2) $$\Upsilon_{B}\left(f\right)=\delta_{B}\left(\epsilon_{B}\left(f\right)\right)$$

In Equation (2), dilation from erosion is defined by $\delta_{B}$ and $\left(\epsilon_{B}\left(f\right)\right)$ Here, $\Upsilon_{B}\left(f\right)$ is the morphological opening.

One-dimensional opening [36] with size u means that all runs of pixels are deleted which are less than u, while the rest of the pixels remain untouched (Algorithm 2).
**Algorithm 2** Morphological opening**1. BEGIN**2.  **VOID** open1d(**image,u**) {3.   **FOR** i in 1,length(image.lines) do4.    **line** = image.lines[**i**]5.    **filtered** = []6.    **FOR** j in 1,length(line.runs) do7.      if runs[j].width() >= u then8.      **filtered**.append(line.runs[j])**9.    ENDFOR**10.    **image**.lines[i] = **filtered****11.   ENDFOR****12. END**

**Figure 6:**
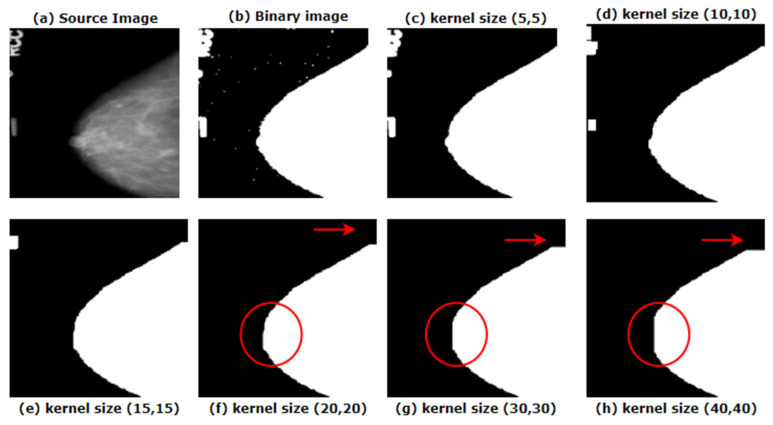
Morphological opening with different kernel sizes.

Morphological opening is applied on the binary image using a kernel. The shape of this kernel depends on the characteristics and the size of the artefacts to be removed. As shown in Figure 6, we have experimented with different kernel sizes to select the most suitable kernel for our images.

**Figure 7:**
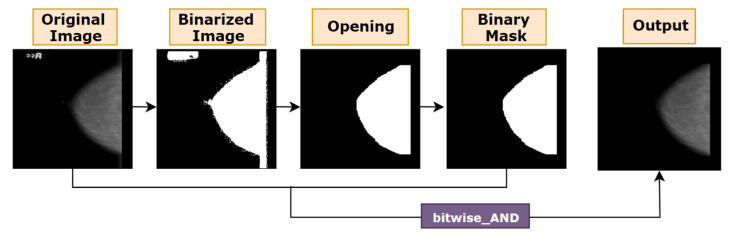
Process flow of morphological opening.

In Figure 6b, after binarizing the original image, small noises become visible which were difficult to found in Figure 6a, the source image. To eliminate tiny noises from image, a small kernel size would be suitable. Figure 6c shows how a small kernel size of (5, 5) removes the noise from the images. However, the artefacts are still present. As the artefacts presented in our image are quite large, the filter or kernel should be large enough to eradicate these artefacts, but not so large that it removes the breast area. Red marked areas of Figure 6f–h show how with an increase of filter size, more pixel information is lost. For the entire dataset, it is found that for a kernel size of (20, 20), results in the optimal outcome. The algorithm is applied using the cv2.morphologyEx function. Along with kernel size, this function requires another parameter, type of morphological operation. As morphological opening is applied, cv2.MORPH_OPEN is used. Thus, the noises are removed from the binarized image and the noise-free mask is then merged with the original image with the help of bitwise_AND function. Figure 7 shows how the artefacts have been removed with the binary mask provided by morphological opening.

#### 5.1.3. Largest Contour Detection

After the completion of morphological opening, some images still contain large artefacts that the morphological opening could not remove. The kernel size could be increased to remove these artefacts. But as described, this approach would result in losing necessary details for most of the mammograms (Figure 6). We therefore use another approach to remove these unwanted objects from the mammograms, largest contour detection. Contour detection algorithms are fundamentally required for performing practical tasks, such as object recognition [37]. Using contour detection, boundaries can be found for every object presented in an image. Using openCV, contours can be detected and regions can be marked with the help of the findContours, and drawContours functions. The function findContours takes three arguments, input image, contour retrieval mode, and contour approximation method. Our first parameter is the breast mammogram (output of morphological opening). The second argument, contouring retrieval mode is passed cv2. RETR_EXTERNAL, as our aim is to retrieve the largest blob. As a third parameter, cv2.CHAIN_APPROX_SIMPLE is used. Rather than retrieving all the coordinates presents in a contour, cv2.CHAIN_APPROX_SIMPLE reserves only the important coordinates. For instance, it does not retrieve all the points of a rectangular shapes contour. Only the four necessary points by which a rectangle can easily be identified and derived are stored, eradicating the redundant points. This approximation method is employed as it saves memory while not compromising the quality of the output. Afterwards, findContours function provides outputs of a python list of all contours and a hierarchy.

**Figure 8:**
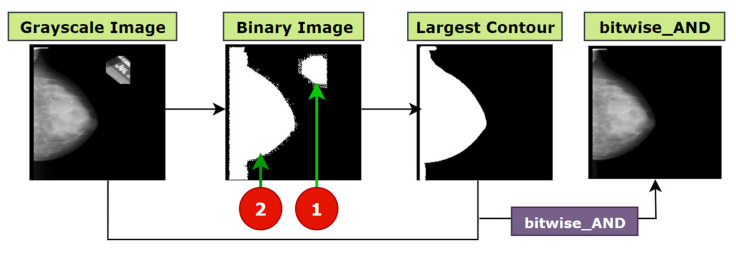
Work flow of largest contour detection.

The drawContours function takes the image and the list of contours as arguments. As our aim is to find the breast, which is larger contour than the artefacts, we will just pass the image, the largest contour and the index of the contour as arguments. Thus, with the help of findContours function we locate all the objects in the mammograms, organize them in descending order and select the largest object. This method requires binary thresholding. By applying binary thresholding, all the objects in the image will be converted to white (1) having same intensity value. The remaining pixels of the image will be converted to black (0) and ignored. All white pixels separated by black pixels will be considered as a contour. As a result, this algorithm can utilize the benefit of binary thresholding while looking for the borders of the objects with similar color intensity. A thing to be noted is that the algorithm would not work properly with RGB (red, green, blue) format because the intensity values of the objects may vary while largest contour detection algorithm requires similar intensity. The findContours function returns a list of all the contours from which the largest one can easily be detected. The max function is used to find the largest contour from the list. Two arguments are passed to the function, contour list and a key, which is cv2.contourArea. Using the key, the function extracts the largest contour from the list. After getting the largest contour, draw Contours function draws the area over it and a binary mask containing only the largest blob is returned. This function requires three parameters, the source image, the list of contours, the index of contours to be drawn, color and thickness. We have passed the mammogram as the source image. In the contour list there is just one contour as max function derives only the largest one. Therefore, as third argument, −1 is passed as the contour index, as −1 refers to all the contours presented in contour list. For color and thickness (255, 255, 255) and 1 are passed respectively. Finally, the original image is merged with this binary mask. The result is shown in Figure 8.

### 5.2. Remove Line

**Figure 9:**
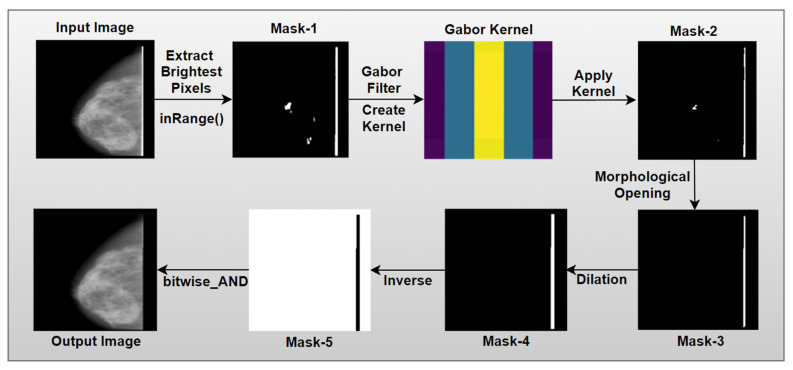
Complete remove line process.

As can be seen from Figure 3, ‘remove line’ is another function to remove artefacts from the image. Some images were found to have a straight bright line attached to the breast contour (Figure 2). For this purpose, some other algorithms are applied and the entire process flow is showed in Figure 9.

#### 5.2.1. InRange Operation

The way in which we can differentiate the line from the actual breast contour is by looking at its properties. The line contains the brightest pixels and it is vertical and straight. Hence, the line removal task involves detecting the brightest pixels of the image which form a vertically straight line. In binary thresholding, the inRange function allows color based segmentation of the desired object. For color based segmentation HSV (Hue, Saturation, and Value) [38] provide better result than RGB (red, green, blue) or any other color space. The following operations are performed to convert an image from the RGB to HSV format [43]:

(3) V←max(R,G,B)

(4) $$S\leftarrow \left\{\begin{array}{cc} V-\frac{\min\left(R,G,B\right)}{V} & \;\mathrm{If}\;V\neq 0\\ 0 & \;\mathrm{otherwise}\; \end{array}\right\}$$

(5) $$H\leftarrow \left\{\begin{array}{cc} \frac{60\left(G-B\right)}{V-\min\left(RGB\right)} & \;\mathrm{If}\;V=R\\ 120+\frac{60\left(B-R\right)}{V-\min\left(RGB\right)} & \;\mathrm{If}\;V=G\\ 240+\frac{60\left(R-G\right)}{V-\min\left(RGB\right)} & \;\mathrm{If}\;V=B \end{array}\right\}$$

With the help of openCV’s inRange function shown in Equation (6), any pixel in this range is kept while the rest is ignored.

(6) $$\mathrm{Output}\;\leftarrow \left\{\begin{array}{cc} 1 & \;\mathrm{If}\;\mathrm{Input}\;\in \mathrm{Range}\;\\ 0 & \;\mathrm{If}\;\mathrm{Input}\;\notin \;\mathrm{Range}\; \end{array}\right\}$$

The InRange function takes the input image, lowest color range and highest color range as arguments and returns a binary mask containing only the pixels that fall within the given range. In our study, the image resulting from the artefacts removal process is converted to HSV and two color ranges are selected for the filter. For the inRange operation, the possible color ranges are between (0, 0, 200) and (255, 255, 255). The lowest color is selected after experimenting with several values. For this tuple, the optimal outcome is achieved as for most of the mammograms, the lowest pixel value is around the (0, 0, 200) value. After applying this method, the bright vertical line is extracted successfully. However, along with this a portion of the breast has also been extracted in mask-1 shown in Figure 9.

#### 5.2.2. Gabor Filter

If mask-1 (shown in Figure 9) is used to remove the line from images, then some breast part will be eliminated as well. Gabor is a linear filter which is widely used in texture analysis, feature selection and extraction in image processing [39]. A two-dimensional Gabor kernel can visualize an image in such a way that any shape, size and angle of particular object can be identified. This filter resembles the human brain’s visual system and can carry out complex feature extraction which is difficult to accomplish for normal filters. Gabor filters are band pass filters, which mean they perform with in a certain band of frequencies. Let the Gabor function *G* have the parameters: *x*, *y*, $\sigma_{x},\sigma_{y},$ *σ* and *φ*. Here, *x* and *y* are the size of the two-dimensional kernel, $\sigma_{x}\;\mathrm{and}\;\sigma_{y}$ (sigma) are the standard deviation in the *x*- and *y*- directions respectively, *φ* denotes the orientation of the filter and *λ* (lambda) typically represents wavelength. A 2-D Gabor filter is a complex sinusoidal plane modulated by a Gaussian function in the spatial domain [44].

(7) $$h\left(x,y,\lambda ,\phi ,\sigma_{x},\sigma_{y}\right)=\frac{1}{2\pi \sigma_{x}\sigma_{y}}exp\left\{-\frac{1}{2}\left(\begin{array}{c} \frac{R_{1}^{2}}{\sigma_{x}^{2}}\\ +\frac{R_{2}^{2}}{\sigma_{y}^{2}} \end{array}\right)\right\}exp\left\{i\frac{2\pi R_{1}}{\lambda }\right\}$$

where, $R_{1}$ and $R_{2}$ are

(8) $$R_{1}=x\;cos\;\emptyset +y\;sin\;\emptyset$$

(9) $$R_{2}=y\;cos\;\emptyset -x\;sin\;\emptyset$$

In the frequency domain, the Gabor filter is defined as follows:

(10) $$H\left(u,v,\lambda ,\emptyset ,\sigma_{x},\sigma_{y}\right)=Kexp2-2\pi^{2}\sigma_{x}^{2}{\left(\;F-\frac{1}{\lambda }\right)}^{2}+\sigma_{y}^{2}\;F_{2}^{2}$$

where K is a constant, and $F_{1},F_{2}$ are defined as:

(11) $$F_{1}=u\;cos\;\emptyset +v\;sin\;\emptyset$$

(12) $$F_{2}=u\;cos\;\emptyset -v\;sin\;\emptyset$$

By changing the parameters above, filters can be created to match the region that should be extracted. For instance, if the features are large, the sigma value should be large and vice versa. The value of *φ* is changed to get the features in horizontal, vertical, diagonal, or any other direction. The wavelength can be minimized or maximized by changing the value of lambda. For pseudo code of the Gabor filter, see Algorithm 3 [44].
**Algorithm 3** Pseudo-code of Gabor feature extraction**1.  BEGIN**2.   **FOR** each frequency f in the frequency list **f_list**:**3.    FOR** each orientation φ in the orientation list o_list **DO**4.     Construct a gabor filter **g(f, φ)**,5.     Convolve **g(f, φ)** with original image **I**, get response image **R**,6.     Compute the mean **(m)** response in **R**,7.     Count the # of pixels **Np** that have a larger value than **m**,8.     Divide **R** into **X x Y** frames,9.     **FOR i** in range**(1,X)**;10.      **FOR j** in range**(1,Y)**:11.       Count the # of strong response **Ni,j** and Compute the ratio **r**:**12.       r = Ni,j/Nr**;**13.**       Append r to the feature vector **z**:**14.**   Finally, z = [r1,r2,…r|f_list|*|o_list|*X*Y].**15.  END**

In this case, Gabor filter is applied using cv2.getGaborKernel function which requires six arguments: ksize, sigma, theta, gamma, lambd, psi, and ktype. Here, ksize is the size of the Gabor filter or kernel, sigma is the standard deviation value for Gaussian function, theta is the orientation of the Gabor function, gamma is the spatial aspect ratio, lambd is the wavelength, psi is the phase offset, and ktype is the filter type. By changing these values, a suitable Gabor kernel can be formed of several shapes and orientations, based on the characteristics of the dataset and the target application. For our dataset, a Gabor kernel is created with a kernel size of (5, 5), sigma of 3, theta of 1*np.pi/1, lambd of = 1*np. Pi/4, gamma of 0.5, phi of 20 and ktype of cv2.CV_32F. After creating the kernel, using cv2.filter2D function, the Gabor filter is applied on the image. Here, Gabor filter is applied on the resultant image after extracting brightest pixel (mask-1). The resulted image after applying Gabor filter is denoted as mask-2 as shown in Figure 9.

#### 5.2.3. Morphological Operations

Morphological operations are typically used to eliminate imperfections from an image during pre-processing [45]. In this section, morphological opening and dilation to generate the final mask is described.

##### Opening

After the previous step, some noise is still present in Mask-2 (shown in Figure 9). To overcome this issue, morphological opening is performed again. As stated, the kernel or filter size depends on the operation to be performed. In this step, to apply morphological opening, the cv2.getStructuringElement function is used to create a rectangular kernel (1, 30) to extract the straight vertical line while ignoring every object with a height below 30. Thus, the noise in mask-2 is removed, resulting in an output denoted as mask-3 as shown in Figure 9.

##### Dilation

Dilation, represented by the symbol of ⊕, is a morphological operation that adds pixels to the boundaries of foreground objects in order to expand them [46]. A given kernel decides how many pixels are to be added to emphasize the size of the foreground object. Equation (13) is used in this regard.

(13) $$X⊕B=\underset{b\in B}{\cup }X_{b}=\underset{x\in X}{\cup }B_{x}=\left\{x+b|x\in X,b\in B\right\}$$

where X is the input image, B is the given kernel and ⊕ is the symbol of addition [40]. As the vertical line in mask-3 is perceived to be too thin, we have dilated it by a dilation operation. In this step, morphological dilation with a kernel size of (5, 5) is applied using cv2.dilate function to expand the area of the foreground object of a vertical straight line. The resultant image, mask-4 is shown in Figure 9, where the line is more visible.

#### 5.2.4. Inverse Mask

In order to eliminate the vertical line from the original mammogram, this mask-4 needs to be inversed. Hence, mask-4 is inverted by cv2.bitwise_NOT function so that all the pixels except the vertical line of the input image are considered as 1. Equation (14) shows how the pixels of a mask are inverted by subtracting every pixel of the mask from 255. Here, *inv_im* denotes the mask after inversion and *im* denotes the mask before inversion.

*inv_im = 255 − im* (14)

The black pixels of mask-4 will be transformed in white and white pixels in black, resulting in mask-5 as show in Figure 9. Finally, the breast part of the input images is extracted by applying bitwise_AND operation to merge the input image and mask-5.

### 5.3. Image Enhancement

Details of a mammogram are often indistinct which makes it difficult for a model to analyze them. By adjusting brightness, contrast, and smoothness, the quality of an image can be improved. Brightness is a relative term defined as the overall lightness or darkness of an image. Contrast, on the other hand, is the difference in brightness between the object and the background presented. For instance, a bright object on a dark background can be detected more accurately than a dark object on a dark background.

In mammography images, fatty tissues appear dark in a mammogram while both dense breast tissues and tumors have a light appearance. So, even a small tumor can easily be detected in fatty tissues but in dense breast tissues, even large tumors are difficult to detect. Almost 70% of the mammograms of our dataset contain dense tissues. For this reason, good accuracy in detecting tumors cannot be expected without enhancing the image quality. The processes and sub processes of image enhancement are briefly described in this section.

#### 5.3.1. Gamma Correction

Gamma correction, also known as ‘Power Law Transform’, works by controlling the brightness of an image as well as emphasizing the transition of values from dark to light and vice versa by using a nonlinear transformation. By altering this gamma value, the optimal brightness can be acquired in order to detect objects more accurately [41]. With increasing gamma values, dark pixels of an image become darker and bright pixels become brighter. The effect is not the same for all pixels as it balances the overall light and dark tone. As our aim is to highlight the breast lesion on a dark background, gamma correction is adopted for the better distribution of light and dark areas. In this method, the first step requires rescaling the color intensity range from [0, 255] to [0, 1.0]. In Equation (15) for the gamma correction, m is the input mammogram, g is the gamma value, and o refers to the output image.

o = m ^ (1/g)(15)

where, g > 1 means brightening the details.

g = 1 means no effect.
g < 1 means darkening the details.

A power law transformation [47] is denoted by Equation (16), where *x* and *y* are the coordinates of the image:

(16) $$g\left(x,y\right)={\left[f\left(x,y\right)\right]}^{\gamma }$$

**Figure 10:**
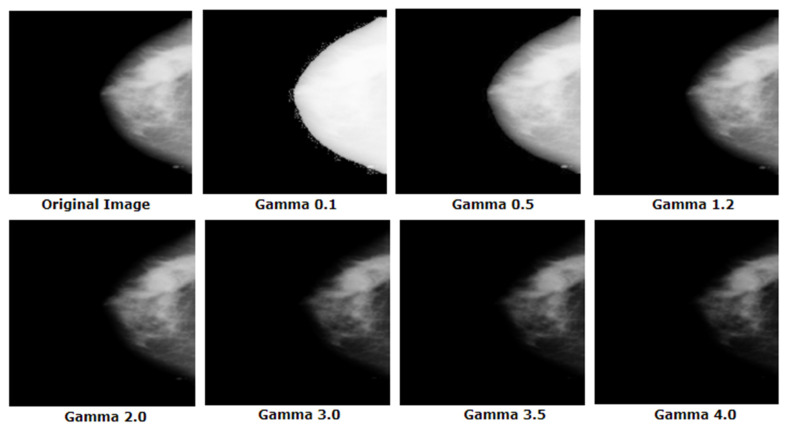
Experimental results with different gamma value.

By experimenting with different gamma values for our images, a suitable gamma value of 2.0 is found which is shown in Figure 10.

**Figure 11:**
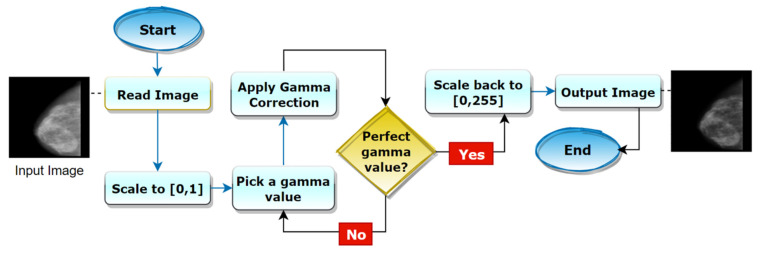
Gamma correction process.

For gamma > 2, the image becomes darkened and pixel information is being lost. For gamma < 1, the image is found faded. In both cases, cancerous tumors are less visible which might lead to poor performance of the CNN. A suitable gamma value can be selected by experimenting with a few different values. A universal value of 2.0 would not be suitable for all datasets, but it is suitable in this case. Finally, the output is scaled back to the original range [0, 255]. Figure 11 shows the procedure of employing the gamma correction process.

#### 5.3.2. Results of the First CLAHE

By using gamma correction, the overall brightness of the image is balanced. Afterwards, to balance the overall contrast, the ‘Contrast Limited Adaptive Histogram Equalization (CLAHE)’ method is used. CLAHE is an advanced variant of Adaptive Histogram Equalization (AHE). Ragab et al. [4] outlined the comparison between these two algorithms and explained why they chose CLAHE over AHE. CLAHE was developed for medical imaging to improve the quality of complex structures [39]. CLAHE enhances contrast locally and improves the readability of medical images [40].

The mathematical equations and formula of CLAHE are briefly explained by the author of this study [42]. Let the size of an image be N×N, and the size of each tile for that image n×n. Then total number of tiles is calculated by using Equation (17),

(17) $$T=\frac{N\times N}{n\times n}$$

The histograms for these tiles are constructed by computing clip limit *C_L_ = N_CL_ × N_AVG_* where *N_CL_* is the normalized contrast limit and *N_AVG_* is the average number of pixels. The average number of pixels is calculated by applying Equation (18),

(18) $$N_{AVG}=\frac{Nx\times Ny}{Ng}$$

where *N_g_* is the number of gray levels for each tile and *N_x_* and *N_y_* are the number of pixels in the *x* and *y* dimensions of the tile. For a gray level j, the frequency *n_j_* exceeds *C_L_*, where *C_L_* represents the clipped pixels. Let, the number of clipped pixels be *N*Σ*_cl_*. The average of the clipped pixels can then be calculated by Equation (19),

(19) $$N_{cp}=\frac{N\sum^{\;}cl}{N_{g}}$$

This is distributed to each corresponding gray level. The number of pixels left to redistribute can be found by using Equation (20),

(20) $$N_{r}=\frac{N_{g}}{N_{r}}$$

where *N_r_* is remaining number of clipped pixels. Thus, the distribution is complete and the contrast limited histogram of a tile is achieved. After performing CLAHE on these tiles, artefacts known as artificial boundaries appear in the image. To eliminate these neighborhood tiles are combined using bi-linear interpolation. For mammogram b(x,y), where *X × Y* is the size of the image and L is the maximum intensity level, the CLAHE processed image is obtained by the following formula:

(21) $$I_{c}\left(x,y\right)=T\left(l\left(x,y\right)\right)=\frac{\left(L-1\right)}{\mathrm{XY}}\overset{K}{\underset{j}{\sum }}n_{j},$$

The CLAHE algorithm has also been described in [42] (Algorithm 4).
**Algorithm 4** CLAHE**Input:** Image after gamma correction **I**;1.  Resizing **l** to N × N; Decompose l—**(n)** tiles; **(n)**—N × N/n × n2.  **H_m_**—histogram(**m**); // histogram of a **n × n** tile;3.  Clip limit: **C_L_—N_cl_ × N_avg_** of **H_n_** using **C_L_**;4.  pixels—distribution over the remaining pixels;5.  **CLAHE(n)**—equalization of contrast limited the histogram tile histogram for the image(l)6.  **b_c_**—bilinear interpolation of CLAHE**Output: CLAHE processed image b_c_**

**Figure 12:**
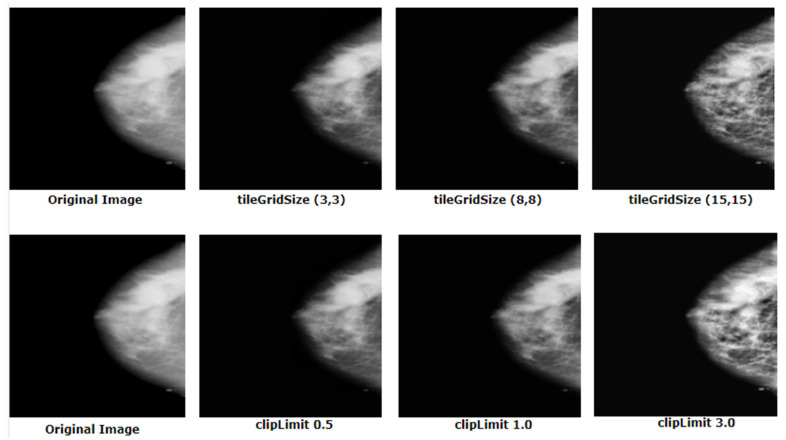
Experimental outcome of CLAHE based on different tileGridSize and clipLimit values.

CLAHE is applied using the cv2.createCLAHE function which requires two parameters, clipLimit and tileGridSize. We have applied a few values of these parameters and selected the ones which led to the optimal outcome. The results are shown in Figure 12.

#### 5.3.3. Outputs after Applying CLAHE a Second Time

As can be observed from Figure 12, with increasing tileGridSize and clipLimit values, necessary pixel information was lost. To distinguish tumors from dense tissue, contrast enhancement is crucial. For this reason, CLAHE is applied again with the same clipLimit value and tileGridSize and it is found that the mammograms are now perfectly enhanced without the loss of any important information. Applying CLAHE twice enhances the visibility of the tumor which results in a higher accuracy. This approach may also be applicable for mammogram like datasets where differentiating the ROI from surrounding tissues is quite challenging.

#### 5.3.4. Green Fire Blue

**Figure 13:**
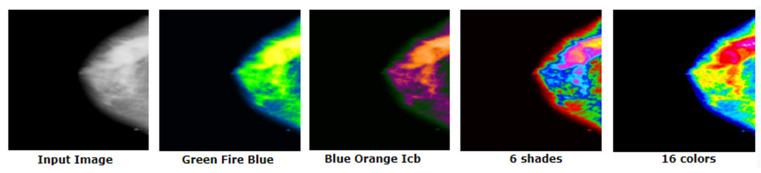
Experimental outcome based on different imageJ filters.

The final enhancement process uses the ImageJ software tool called “Look Up Tables (LUTs)” [9]. This filter is applied on the image to separate the tumor, dense tissues, and surrounding cells in three different colors. There are several filters associated with LUT. We experimented with the filters to find the most suitable one for our dataset. ‘Green Fire Blue’ (GFB) is the LUT filter which worked best in our case. The output of the experimental results with different filter is shown in Figure 13.

Figure 13 illustrates that, for GFB, the cancerous lesion can be better distinguished compared to other filters. However, the other filters have also been assessed as shown in Section 8.3.5.

**Figure 14:**
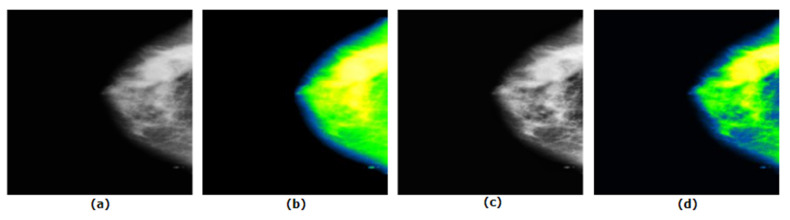
Experimental result of ‘green fire blue’ filter (GFB) based on applying CLAHE once and twice. (**a**) CLAHE 1st, (**b**) GFB applied on CLAHE 1st, (**c**) CLAHE 2nd, (**d**) GFB applied on CLAHE 2nd.

In addition, the GFB filter produces the desired outcome on the mammograms after applying CLAHE second time which is illustrated in Figure 14.

Figure 14 demonstrates that when GFB is applied on CLAHE once, along with the expected tumorous lesion, surrounding dense tissue turns into similar pixel values. When GFB is used after applying CLAHE twice, the tumorous lesion, surrounding dense tissue and other cells are perfectly separated into three different pixel values.

**Figure 15:**
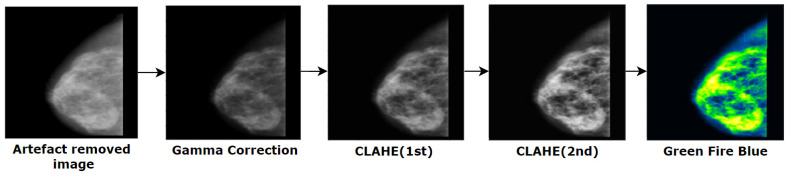
Resulting images after applying Gamma correction, CHALE and Green Fire Blue effect.

Figure 15 illustrates how a cancerous portion is highlighted gradually after every image processing step.

For a better understanding, the parameter value selected for all algorithms based on the considerations described above are given in Table 2.

Moreover, as a part of an ablation study, the impact of the parameter values and choosing suitable algorithms for these pre-processing steps on the model’s performance is described in Section 8.3.6.

### 5.4. Verification

Several algorithms have been used for image processing which may effect on image quality. Statistical analysis is performed to ensure that the image quality is not reduced. We have computed the value of PSNR, MSE, SSIM, and RMSE [9], and generated histogram plots by comparing original images and the ‘Green Fire Blue’ enhanced images.

#### 5.4.1. MSE

MSE defines the cumulative squared error between the pixels of the two images to be compared. It provides an estimate of the image quality. The range of MSE value is between 0 and 1 where a value close to 0 means good image quality. Values greater than 0.5 mean that the quality has been decreased. A value of 0 indicates that image is noise free.

(22) $$MSE=\frac{1}{pq}\sum_{i=0}^{m-1}\sum_{j=0}^{n-1}{\left(O\left(m,n\right)-P\left(m,n\right)\right)}^{2}$$

where *O* is the ground truth image (i.e., original image), *P* is the processed image, *p* and *q* denotes the pixels of *O* and *P,* and *m*, *n* denotes the rows of the pixels *p*, *q.*

#### 5.4.2. PSNR

PSNR denotes the ratio of the maximum possible power of a signal to the power of the corrupting noise affecting the quality of a processed image. For the calculation of PSNR, first the value of MSE is calculated. Then PSNR is derived using this formula:

(23) $$PSNR=20\;{log}_{10}\left(\frac{\left(MAX\right)}{\sqrt{MSE}}\right)\;$$

where MAX is the maximum value of pixels of the image (i.e., 255). For an 8-bit image, a good PSNR value lies usually between 30 to 50 dB [9].

#### 5.4.3. SSIM

SSIM measures the reduction of image quality caused by pre-processing algorithms. The estimation is in the range of −1 to 1, where 1 denotes ‘perfect structural similarity’ and 0 denotes ‘no similarity’ [9].

(24) $$SSIM\left(x,y\right)=\frac{\left(2\mu_{x}\mu_{y}+c_{1}\right)\left(2\sigma_{xy}+c_{2}\right)}{\left(\mu_{x}^{2}+\mu_{x}^{2}+c_{1}\right)\left(\sigma_{x}^{2}+\sigma_{y}^{2}+c_{2}\right)}\;$$

where *µx* and *µy* are the averages of two images (*x*, y), calculated by using the Gaussian window. $\sigma_{x}^{2}$ and $\sigma_{y}^{2}$ denote the variance, $\sigma_{xy}$ is the covariance of the images. $c_{1}$ and $c_{2}$ are the two variables utilized in order to stabilize the division, where *c1* i (0.01 × 255)2 and *c2* = (0.03 × 255)2 and 0.01 and 0.03 are default values.

#### 5.4.4. RMSE

RMSE measures the difference of quality between the original image and the processed image. A lower RMSE, especially values close to 0 means less errors and good image quality.

(25) $$RMSE={\left[\overset{N}{\underset{j=1}{\sum }}{\left(d_{f_{i}}-d_{d}\right)}^{2}/N\right]}^{\frac{1}{2}}\;$$

where ∑ represents summation, ${\left(d_{f_{i}}-d_{d}\right)}^{2}$ is the square of differences and N is the size of the dataset. Values for ten images, selected at random are shown in Table 3.

#### 5.4.5. Histogram Analysis

**Figure 16:**
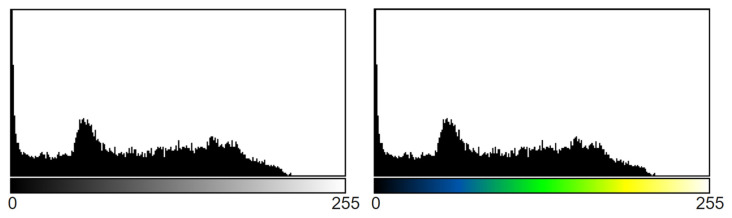
Histogram of original image vs. green fire blue.

A histogram of an image refers to the distribution of intensity of the image by generalizing the pixels having specific intensity levels. This analysis is considered a statistical representation of an image where the intensity level can vary from 0 to 255. It is a graph where the x-axis denotes the intensity value and the y-axis denotes the number of pixels. The resemblance of two images can be visualized using histogram plots of pixel intensity. It is imperative that the histograms of processed image and original image remain similar after image processing; otherwise the key features might be lost due to processing which may cause poor interpretation of model. We adopted histogram analysis method in our study to investigate whether the features of the original mammogram remain unchanged in the processed mammogram. Figure 16 shows the histogram analysis of an artefacts removed mammogram and a processed mammogram. The histogram of artefacts removed image is presented on the left and the histogram analysis of processed image after applying green fire blue on the right. The original artefact removed image was in gray scale format and the Green fire blue enhanced image is in RGB color format. Green fire blue assigns three shades of colors, green, yellow, and blue to each pixel of the gray scale image based on pixel intensity level. As can be seen from Figure 16, despite the different color specification, the histogram analysis shows similar pixel intensity levels for both images. The specific pixels in both images have nearly similar intensity level and it can therefore be concluded that no significant image information was lost due to image processing.

## 6. Data Augmentation

In this section, data augmentation techniques are discussed. Data augmentation techniques are used to enlarge our dataset before splitting the dataset into separate datasets for training, testing and validation. After splitting, the training dataset is utilized to train our transfer learning models.

### 6.1. Data Augmentation

**Figure 17:**
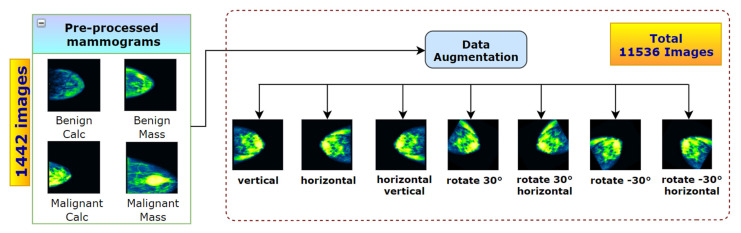
Seven augmentation techniques used to increase the number of images.

To expect good outcome from a deep learning model, 1442 images are not sufficient. Data augmentation techniques [48] can result in an improvement in accuracy. Data augmentation techniques are also a well-known strategy to expand the diversity of the dataset, improving the robustness of the model and contributing to the overall prediction accuracy which has been proven its worth in the field of deep learning. This technique can reduce overfitting in CNN models [49] as the model will have enough distinct data to train on and can also accelerate convergence. The most commonly applied data transformation techniques are flipping, rotating, zooming, mirroring, and cropping. We employ seven augmentation methods on the pre-processed dataset: (1) vertical flipping, (2) horizontal flipping, (3) horizontal-vertical flipping, (4) rotating 30°, (5) rotating 30°-horizontal flip, (6) rotating −30°, (7) rotating −30°-horizontal flip that are shown in Figure 17. A dataset of 11536 mammograms is generated by augmenting the original dataset of 1442 images eight times. The data augmentation process is illustrated in Figure 17.

### 6.2. Data Split

**Figure 18:**
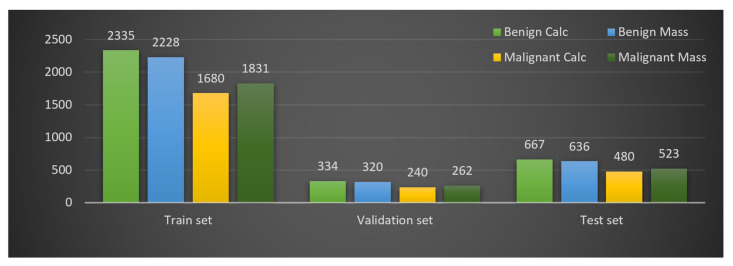
Class distribution of train, validation and test set after splitting augmented dataset.

The next and last step before training involves splitting the dataset. To assess the impact of training–testing data size on the model’s overall accuracy, three splitting ratios for training–testing data (90:10, 80:20, and 70:30) are commonly used [25]. In a recent study [22] 20% images of the dataset were used as test dataset to make the final prediction. We split the mammograms into three sets with a ratio of 70:10:20 for training set, validation set and test set respectively. After splitting the augmented dataset of 11536 mammograms we get 8074 mammograms in the training set, 1156 mammograms in the validation set and 2306 mammograms in the testing set. The mammograms distribution for the four classes in the training, testing and validation sets are shown in Figure 18. The validation set is kept smaller than test set. As validation data are evaluated in every epoch during the training phase, the computational time of the epochs will increase for a larger validation set.

## 7. Proposed Model

As described earlier, to find the best transfer learning model for our classification problem, we experimented with a total of six state of the art models to determine the optimal network based on accuracy. During the training phase, only the weights of the last FC layer of every model were modified and updated. We have created a transfer learning method by adding one flatten and dense layer at the bottom of the pre-trained models. The models are fine-tuned by adding a flatten layer right before the FC layers and changing the output layer with four neurons.

### 7.1. Transfer Learning and ImageNet

Transfer learning refers to reusing a pre trained model on a different problem. In other words, transfer learning is the process of using the weights of a pre-trained model which was trained on a large dataset to a smaller dataset in order to improve the accuracy. Deep learning requires a large number of labelled images (i.e., ImageNet) to train for a given classification problem from scratch [50]. In many cases, sufficient data are not available in the medical imaging domain, and obtaining datasets as detailed as ImageNet remains a challenge [51]. Unfortunately, this also applies to publicly available, breast cancer datasets. To deal-with this concern, transfer learning and fine-tuning can help to increase the accuracy of classifiers by transferring knowledge from another domain where large datasets are available [49]. Transfer learning of a CNN model that is pre-trained with a large scale natural image dataset (i.e., ImageNet), to medical images [51] can be a promising approach. The performance of a fine-tuned network is not much reduced even when the size of training data is quite small [25]. Medical images (i.e., mammograms) may greatly differ from the natural images (i.e., ImageNet), but pre-trained knowledge can be utilized to attain significant improvement of the model’s performance [52] as the pre-trained model’s weight parameters have already been trained to detect primal features such as edges, corners and textures. This method has proven to be very effective in dealing with small data sets. A definition of transfer learning [53], is as follows: in order to improve the performance of a target training model $F^{T}$ over a target domain $D^{T}$ (i.e., processed mammograms) for the evaluation of a target learning task $T^{T}$ (breast cancer classification) by using the pre-trained weights of a source learning task $T^{s}$ (i.e., imageNet) of a given source domain $D^{s}$ (i.e., natural images):

(26) $$D^{s}\neq D^{s}\;$$

(27) $$T^{s}\neq T^{s}$$

The knowledge of $D^{s}$ and $T^{s}$ is used for $D^{T}$ and $T^{T}$. In our case, $D^{T}$ (processed mammograms) are quite different from $D^{s}$ (natural images). But the visual properties of the ROI (region of interest) of any image are quite similar generally (i.e., edges, textures, shapes, etc.) [49]. For pre training, the weights of the ImageNet dataset are used. ImageNet is a wide-ranging dataset consisting of over 14 million 256 × 256 labelled natural images in more than 1000 classes which is publicly available. A model trained with this database, can be used as the foundation for various object detection and ROI segmentation problems especially in smaller datasets of medical image classification problem [51]. Comprehensive annotated medical image dataset equivalent to ImageNet is challenging to get as data collection is laborious, and quality assurance is expensive. In this regard, transfer learning from ImageNet to CAD problems has shown some promising results [54].

### 7.2. Transfer Learning Model and Finetuning

A total of six pre-trained models: InceptionV3, MobileNetV2, ResNet50, DeneNet201, VGG16, and VGG19 are trained on the training dataset and evaluated using the testing dataset to find the best performing model among them.

#### 7.2.1. MobileNetV2

MobileNetV2 is proposed by Google community. It consists of two types of blocks and each block contains three layers. In both blocks the first and third layers are 1×1 convolutional layer with 32 filters and the second layer is a depthwise convolutional layer. Rectified linear activation function (ReLU) is used in all layers. Longitudinal bottlenecks situated between the layers play a key role in preventing non-linearity from damaging a large amount of data. The difference between the two blocks is in stride size as block 1 has stride of one and block 2 has stride of two.

#### 7.2.2. ResNet50

A combination of convolution filters with multiple sizes is used in the ResNet50 architecture to tackle the deterioration issue of CNN models and reduce the training time caused by deep structure. ResNet50 is made up of a total of 48 convolutional layers along with a maxpool layer and an average pool layer. This architecture has over 23 million trainable parameters.

#### 7.2.3. InceptionV3

InceptionV3 focuses on reducing the required computing power by altering prior Inception designs. Factorized convolutions, regularization, dimension reduction, and parallelized calculations are some of the strategies used in keeping the computational cost low. InceptionV3 has some key updates compared to earlier inception models, including label smoothing, factorized 7×7 convolutional layers and the usage of an auxiliary classifier to send label information down the network. The training time is lessened in InceptionV3 model by replacing larger convolutions with smaller convolutions.

#### 7.2.4. DenseNet201

The primary objective of this model is to achieve higher accuracy with fewer parameters. Each layer in DenseNet receives extra input from all prior levels and passes on its own feature-maps to all following layers. Each layer uses the previous layers feature maps. This allows the network to be thinner and more compact, resulting in fewer channels. In other words, each layer accepts the feature maps of the previous layer as input, and the feature maps of the subsequent levels convey all the data by explicitly connecting all the layers in the network.

#### 7.2.5. VGG16

VGG16 is a DCNN model proposed by Simonyan and Zisserman [55]. The model achieved 92.7% top 5 test accuracy in ImageNet dataset and it won the Large-Scale Visual Recognition Challenge (ILSVRC) competition proposed by the Oxford Visual Geometry Group [17]. The increased depth of the VGG model can aid the kernel to learn more complex features. In research on the effectiveness of transfer learning [25] it was found that a pre trained and fine-tuned VGG16 achieved a much higher accuracy then a fully trained network.

#### 7.2.6. VGG19

VGG19 is a variation of the VGG model which has 19 layers in total. It has three additional FC layers containing 4096, 4096, and 1000 neurons respectively at the end of the VGG16 model making it a total of 19 layers. It also consists of five maxpool layers and a Softmax layer. The convolutional layers are equipped with ReLU activation function.

### 7.3. Training Approach

To train the models, the maximum number of epochs was set to 350 and the batch size to 16 [22]. Optimizer Adam was used with a learning rate of 0.001. The weights of the best model, based on a minimum loss value using ‘callback’ function of Keras [22] were saved during the training process. To analyze the model’s accuracy, we have used Keras’s CSVLogger function which saves a comma-separated values (CSV) file of training and validation performances for every epoch. ‘Categorical cross-entropy’ is used as the default loss function of multi class problems [56]. As mentioned earlier, ‘*Softmax*’ activation is used for the prediction of probability for each class. Softmax normalizes all the values in the range of 0 to 1 and the summation of these values always equals 1. If the probability of class changes it affects the values of the probabilities for the other classes so that the summation of probabilities does not change.

(28) $$softmax\left(z_{i}\right)=\frac{exp\left(z_{i}\right)}{\Sigma_{j}exp\left(z_{i}\right)}$$

The mathematical expression of the Softmax function is described in Equation (28), where $z_{i}$ refers to the outputs of the output neurons and the inputs of the Softmax function. Exp () is a non-linear exponential function which is applied to each value of $z_{i}$. The bottom part of the Equation (28) ($\Sigma_{j}exp\left(z_{i}\right)$) normalizes the exponential values by dividing them by the summation of $exp\left(z_{i}\right)$. As we have experimented with multiple models and configurations, three computers were utilized equipped with Intel Core i5-8400 Processor, NVidia GeForce GTX 1660 GPU, 16 GB of Memory, and 256 GB DDR4 SSD for storage.

### 7.4. BreastNet18

**Figure 19:**
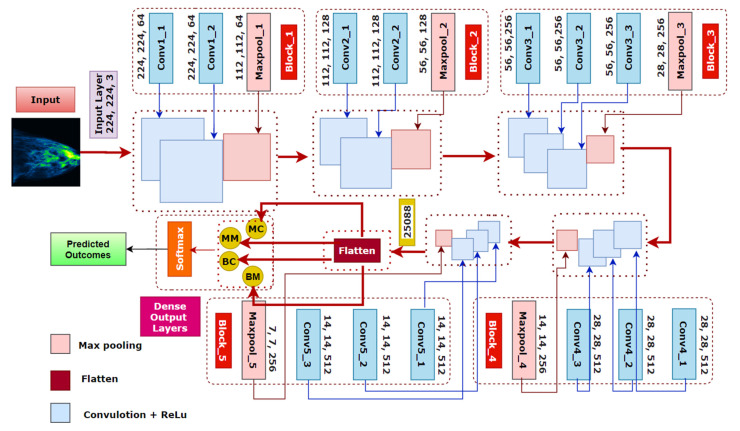
Architecture of BreastNet18, a fine-tuned VGG16 network.

Of the six architectures described above, fine-tuned VGG16 yields the highest classification accuracy. We therefore propose a model, called BreastNet18, which is built upon a fine-tuned VGG16 architecture where several experiments are carried out with our dataset to evaluate the performance. Moreover, an ablation study is carried out to increase the robustness of the architecture for the mammogram classification task. The model’s architecture is shown in Figure 19.

The original VGG16 has a depth size of 23 and, as the name indicates, it has 16 layers. It consists of five blocks and each block contains two or three convolution layers followed by a Maxpooling layer. A convolution layer performs a linear operation by multiplying an array of input data with a two-dimensional array of weights, known as filter or kernel.

(29) $$\left(f*g\right)\left\{n\right\}=\overset{m}{\underset{k=1}{\sum }}g\left(k\right)\cdot f\left(n-k+m/2\right)$$

Here, g( ) and f( ) are the input function and the kernel function and (f∗g) denotes the dot product of these functions over a number of variables n. After obtaining the feature matrix from the convolution layer, the Maxpooling layer decreases the dimensions of the maps by applying filters or kernels. For an input image size of (m×m) and a filter size of ((f×f)), the output size after applying the filter on the input matrix will be:

(30) (m×m)∗(f×f)=(m−f+1)×(m−f+1)

Our modified model BreastNet18 is generated by adding a flatten and a dense layer after the fifth block of VGG16. As by default the input layer of the architecture requires that the size of the image, which is an RGB image, is 224 × 224 × 3, our input dimension for the first convolutional layer is 224 × 224 × 3. In this context, the first block contains two convolutional layers with 64 channels of 3 × 3 kernel size and the same padding followed by a 2 × 2 Maxpooling layer of stride 2 × 2. Similarly, the second block contains two convolution layers of 128 channels, with a kernel size of 3 × 3, followed by a Maxpooling layer of stride 2 × 2 as in block one. The last three blocks contain three convolutional layers followed by a Maxpooling layer. The channel sizes of the three convolutional layers in blocks 3, 4 and 5 are respectively 256, 512 and 512, all having the same kernel size of 3×3. The initial input image is shrunken to half the size in each Maxpooling layer. After the stack of convolutional and Maxpooling layers the outputted feature map from the last Maxpooling layer is of size 7 × 7 × 512. A flatten layer has been added to make a 1 × 25,088 feature vector. A dense layer has also been added which outputs four channels for the four classes. There is a Softmax activation function at the end which normalizes the classification vector obtained from FC. As shown in Figure 19, the light blue boxes in five blocks represent convolutional blocks which are imported from the VGG16 model, pre-trained on the ImageNet database.

**Figure 20:**
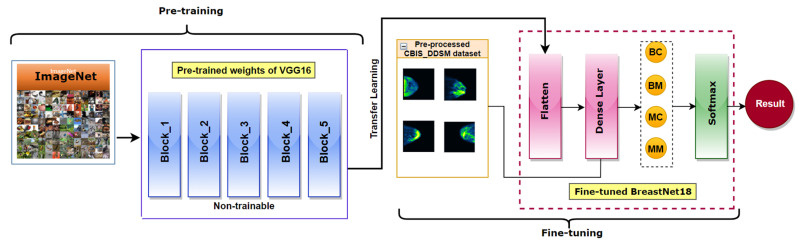
Fine-tuning process of a pre-trained VGG16 model.

As explained above, our proposed BreastNet18 model is obtained by the addition of one flatten and one dense layer after the fifth block of the VGG16 network. As shown in Figure 20, there are two phases: pre-training and fine-tuning. In the pre-training phase, during the training process of our experiment, the weights of the layers remain frozen. The purple boxes represent the flatten layer and the dense layer of BreastNet18 which are updated by the new features of our mammograms in the fine-tuning phase. The last FC layer is modified to classify four classes instead of 1000 classes. Thus, the pre-trained weights of VGG16 have been transferred using the transfer learning method and the model was then retrained with our mammogram dataset. Finally, the activation function ‘Softmax’ calculates the probabilities for each of the classes and generates the predicted outcome.

### 7.5. Ablation Study

In CNN based applications, an ablation study is commonly performed to examine the stability and performance of the model after removing or altering different layers and hyper-parameters. When these components are changed within the architecture of a model, the network can show identical, increased, or decreased performance. Mostly, by experimenting with different hyper-parameters such as optimizer, learning rates, loss functions, and batch sizes, the accuracy can be boosted. Changing the architecture of the model also impacts on the overall performance. In this research, various configurations of our proposed BreastNet18 model are investigated by randomly eliminating or modifying different components and parameters. In this regard, four case studies are performed and the findings analyzed. Results suggest that the overall accuracy is increased which proves the effectiveness of this technique for the study of DCNN. The results of this ablation study are described in Section 8.3.

## 8. Results and Discussion

### 8.1. Evaluation Matrix

In order to evaluate the transfer learning models, we have used several metrics: precision, recall, F1-score, accuracy (ACC), sensitivity, and specificity. A confusion matrix was generated for all the models. From this we get the values of true positive (TP), true negative (TN), false positive (FP), false negative (FN) cases. In some cases, area under curve (AUC) value was calculated. Furthermore, false positive rate (FPR), false negative rate (FNR), and false discovery rate (FDR), mean absolute error (MAE), and root mean squared error (RMSE) was calculated for doing statistical analysis of the models.

(31) $$\mathrm{ACC}=\frac{\mathrm{TP}+\mathrm{TN}}{\mathrm{TP}+\mathrm{TN}+\mathrm{FP}+\mathrm{FN}}$$

(32) $$\mathrm{RecaIl}=\frac{\mathrm{TP}}{\mathrm{TP}+\mathrm{FN}}$$

(33) $$\mathrm{Specificity}=\frac{\mathrm{TN}}{\mathrm{TN}+\mathrm{FP}}$$

(34) $$\mathrm{Precision}=\frac{\mathrm{TP}}{\mathrm{TP}+\mathrm{FP}}$$

(35) $$F_{1}=2\frac{\mathrm{precision}*\mathrm{recall}}{\mathrm{precision}+\mathrm{recall}}$$

(36) $$\mathrm{FPR}=\frac{\mathrm{FP}}{\mathrm{FP}+\mathrm{TN}}$$

(37) $$\mathrm{FNR}=\frac{\mathrm{FN}}{\mathrm{FN}+\mathrm{TP}}$$

(38) $$\mathrm{FDR}=\frac{\mathrm{FP}}{\mathrm{TP}+\mathrm{FP}}$$

(39) $$\mathrm{MAE}=\frac{1}{n}\overset{n}{\underset{j=1}{\sum }}\left|y_{j}-y_{j}^{p}\right|$$

(40) $$\mathrm{RMSE}=\sqrt{\frac{1}{n}\overset{n}{\underset{j=1}{\sum }}{\left(y_{j}-y_{j}^{p}\right)}^{2}}$$

AUC value is derived from receiver operating characteristic (ROC) curve which plots the true positive rate (TPR) against false positive rate (FPR) at several threshold values where TPR is another term of Recall. FPR is calculated as:

(41) $$\mathrm{FPR}=1-\mathrm{Specificity}=1-\frac{\mathrm{TN}}{\mathrm{TN}+\mathrm{FP}}$$

### 8.2. Results of Transfer Learning Models

Table 4 shows the results from all six models. It can be seen that the VGG16 model achieved the best test accuracy of 97.13% with a test loss of 0.081. VGG16 also had the highest training and validation accuracy of 97.79% and 96.83% respectively while maintaining a 0.18 training loss and a 0.11 validation loss. VGG19 recorded accuracies close to the best model with 96.63%, 95.63% and 96.24% respectively for training accuracy, validation accuracy and testing accuracy. DenseNet201, a deep model, produced a test accuracy of 86.92%. MobileNetV2, ResNet50 and InceptionV3 all had a moderate performance with accuracies between 75% and 80%. VGG16 recorded a precision of 97.29%, a recall of 97.02% and an F1 score of 97.15%. It also had good sensitivity and specificity, of 98.03% and 98.14% respectively. All these results (Table 4) clearly highlight the classification capabilities of VGG16 and its robust performance on our dataset. We have therefore chosen VGG16 as our base model and subsequently performed various ablation studies on it to make the model more robust for this classification problem.

#### 8.2.1. Statistical Analysis of the Models

Table 5 shows the experimental results of the statistical analysis for the different models. To evaluate the statistical significance of the proposed method compared to other methods, false positive rate (FPR), false negative rate, false discovery rate, Kappa coefficient (KC) value and Matthew’s correlation coefficient (MCC) values are derived for all six transfer learning models. VGG 16 outperformed all the other models with a FPV value of 1.97%, FNR of 2.98% and FDR of 2.71%. VGG19 had a FPV of 2.67%, FNR of 3.37% and FDR of 3.67% which is close to the results of VGG16. On the other hand, InceptionV3 resulted in the poorest outcome with a FPR value of 21.85%, FNR of 23.16% and an FDR of 23.26%. Moreover, regarding KC and MCC, VGG16 provided the best performance achieving the scores of 98.52% and 88.62% respectively. The lowest KC and MCC values were recorded for InceptionV3 with a KC value of 75.81% and an MCC value of 67.02%.

For further comparison, mean absolute error (MAE) and root mean squared error (RMSE) are calculated to assert the effectiveness in terms of error rates which is shown in Table 6. In this regard, the best performing model is the one with the lowest error rate. It is clear from Table 6 that VGG16 had the lowest MAE value (2.44%) with a RMSE value of 7.20%. VGG19 recorded similar error rates with an MAE of 3.16% and an RMSE of 12.51%. Other transfer learning models gave MAE values ranging from 10% to 18% and RMSE values ranging from 25% to 37%.

#### 8.2.2. Comparison among the Models Based on Layer Depth and Trainable Parameters

For a small dataset, a CNN with lower layer depth architecture is preferable to get a good performance while minimizing computational cost. A deep CNN architecture with large number of trainable parameters increases the need to have a large dataset and results in a high computational cost. Often, in publicly available medical datasets, the number of samples is quite inadequate. Therefore, to achieve a good performance from such datasets, using a CNN model with less layer depth can be a better approach. The specifications of the models in terms of layer depth and trainable parameters are shown in Table 7.

As we are experimenting with small number of images, VGG16 with a relatively shallow layer depth of 16 layers and 50,178 trainable parameters is preferable compared to other models. Moreover, this model yielded the highest classification performance with no indication of overfitting.

### 8.3. Results of Ablation Study

As ablation study, four experiments are performed, altering different components of the proposed BreastNet18, based on the fine-tuned VGG16 architecture. By making alterations to various components a more robust architecture with higher classification accuracy can be achieved. For this purpose, an ablation study for various components: Batch size, Flatten layer, Loss function, Optimizer, and learning rate is performed.

#### 8.3.1. Ablation Study 1: Changing Flatten Layer

In Table 8, the optimizer is denoted as ‘OP’, the learning rate as ‘LR’, the validation loss as ‘Val_Loss’, the validation accuracy as ‘Val_Acc’, the test loss as ‘Ts_Loss’, and the test accuracy as ‘Ts_Acc’. A flatten layer is connected to the FC layer that converts 2-dimensional feature maps of the preceding convolutional layers into a 1-dimensional array. In our study, previously a flatten layer was added to flatten the feature maps. This layer is replaced with GlobalAveragePooling2D and GlobalMaxPooling2D to observe its impact on the network’s performance. Table 8 shows that for GlobalAveragePooling2D, the network performs with identical results and for GlobalMaxPooling2D the accuracy drops slightly.

#### 8.3.2. Ablation Study 2: Changing Batch Size

The number of training samples utilized in a single iteration is referred to as batch size. In our study, we have experimented with various batch sizes (Table 9) to find the best batch size for our proposed model. After changing the batch size from 64 to 16 and 32, an increase in both test and validation accuracies is observed (Table 9). While testing with batch size 16, the test accuracy was 97.44%. The highest test accuracy, 97.57%, and the lowest loss, 0.81, are observed with a batch size of 32. As batch size 32 gives improved test accuracy, this batch size is used further experimentation.

#### 8.3.3. Ablation Study 3: Changing Loss Functions

Experimentation with various loss functions, Categorical Crossentropy, Cosine similarity, and Mean Squared Error loss was carried out to find the suitable loss function for our proposed model. Table 10 shows the performance of the model with the selected loss functions. While equipped with Categorical Crossentropy the model had a 97.57% test accuracy which is the best result. Both the Cosine similarity (96.93%) and the Mean Squared Error loss functions resulted in a small decrease in test accuracy (96.72%). To achieve the best classification performance, the Categorical Crossentropy loss function was selected for further experimentation.

#### 8.3.4. Ablation Study 4: Changing Optimizer and Learning Rates

It can be observed from Table 11 that the performance improved with optimizer Adam resulting in the highest test accuracy of 98.02% with a learning rate 0.0008. Adam with learning rate 0.0008 also had the lowest test loss, of 0.05. The highest validation accuracy is 97.03%, achieved with optimizer Adam with learning rate 0.0008. The majority of the models had a reasonable performance with accuracies above 95%.

#### 8.3.5. Ablation Study Based on Different ImageJ Filters

As previously mentioned, we have evaluated the effect of using different filters in the preprocessing stage, see Table 12.

It can be observed from Table 12 that the dataset formed by applying ‘Green Fire Blue’ filter outperforms others with a test accuracy of 98.02% and an F1 score of 98.15%, demonstrating the robustness of applying this filter.

#### 8.3.6. Ablation Study Based on Image Processing Algorithms

As a part of our ablation study, the impact of the applied pre-processing algorithms with suitable parameter values on the model’s performance is also investigated. Results are shown in Table 13. This assesses the effectiveness of employing the pre-processing algorithms before training the model.

It is observed that the accuracy increases gradually for every step, from 79.32% to 98.02%. As described, after applying CLAHE twice, the cancerous lesion is more highlighted, which combined with Green Fire Blue filter results in the best accuracy (98.02%).

### 8.4. Performance Analysis of Best Model

After conducting the ablation study on the proposed BreastNet18 model, an improvement in classification accuracy is observed when the optimal batch size, learning rate and optimizer are used. Table 14 gives an overview of the final configuration of BreastNet18. For this configuration of BreastNet18, the training accuracy curve, validation accuracy curve, training loss curve over the training phase, and confusion matrix were generated.

**Figure 21:**
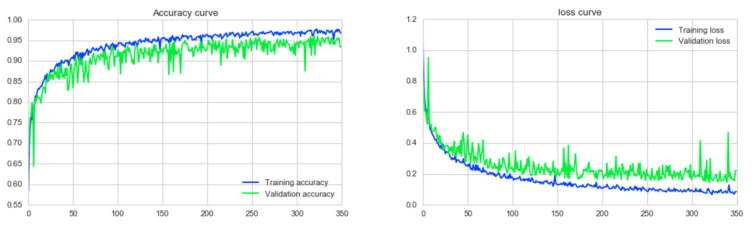
Loss curve and accuracy curve over 350 epochs for Adam with learning rate 0.0008.

In Figure 21, the learning curves for our best performing model are presented, loss curve and accuracy curve. As can be seen, the training curve converges smoothly from the first to the last epoch with almost no bumps. The gap between the validation accuracy curve and the training accuracy curve gives no indication of overfitting during training. Like the training curve, the loss curve shown in Figure 21 converges steadily to the final epoch. In conclusion, after analyzing the training and loss curves, no evidence of over fitting or underfitting was found.

**Figure 22:**
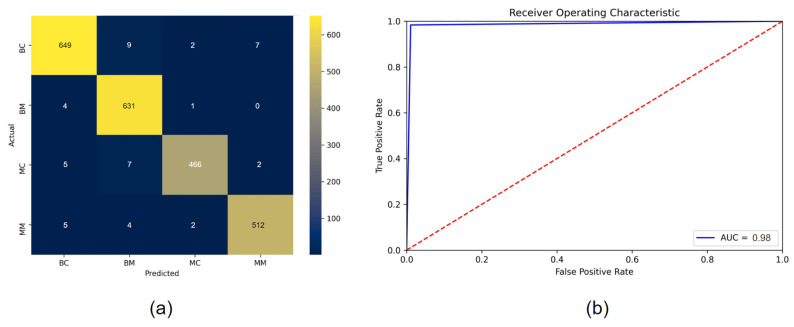
(**a**) Confusion matrix and (**b**) ROC curve of proposed BreastNet18 after ablation study.

Figure 22 a represents the confusion matrix for the model with the highest accuracy. The row values represent the actual label of the test images and the column values represent the predicted label of the test images. The diagonal values are the TP. The confusion matrix of the proposed BreastNet18 model with the optimal configuration based on the ablation study, Adam and learning rate 0.0008, is not biased to any one class and predicts all classes almost equally. However, the model had the best results for Benign Mass (BM).

The receiver operator characteristic (ROC) probability curve is plotted and the area under the curve (AUC) value is derived from the ROC curve. The AUC is used as a summary of the ROC curve and represents the ability of a model to distinguish the different classes. An AUC value close to 1 indicates that the model is able to detect most classes. As can be seen in Figure 22b, the ROC curve almost touched the peak of y-axis which means that the false positive rate is close to 0 and the true positive rate is close to 1. We get an AUC value of 98.27% which demonstrates the effectiveness of the model.

**Figure 23:**
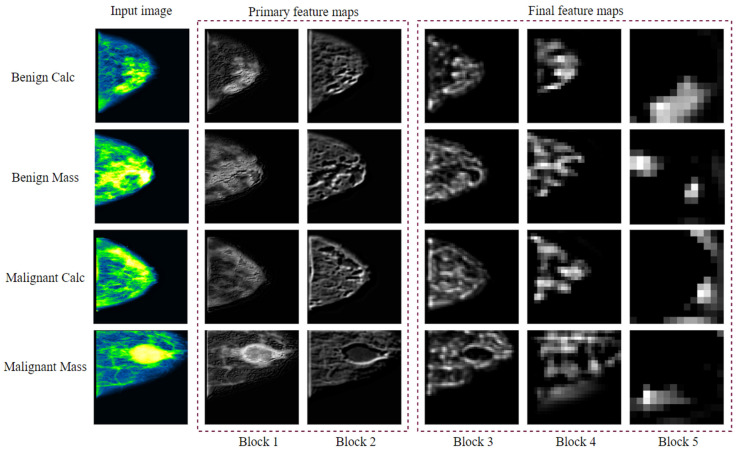
Feature maps of all four classes from 5 blocks of VGG16.

In addition, feature maps generated by the model and the model’s capability to extract abstract features are evaluated. Figure 23 shows the feature maps produced at the end of each Block of BreastNet18. Although the inputted images were in RGB format, the feature maps created are in grayscale format. Figure 23 gives an impression of the learned feature maps for all four classes (BC, BM, MC and MM). As can be observed in the primary feature maps (Figure 23), the first two blocks of the architecture are dedicated to learning distinct textural features while preserving the structural information of the input image. Abstract representations of the images are learned with the deep layers of Block 3, 4, and 5 which can be seen in the final feature maps of Figure 23. The architecture was able to successfully distinguish cancerous features from the images and a clear discriminative pattern can be observed from the feature maps obtained from images of four different classes (BC, BM, MC, and MM). Discriminative feature distribution in the feature maps of the different classes adds to the generalization capability of the model, increasing the performance of the proposed BreastNet18 architecture.

### 8.5. Check on Overfitting

In this work, we have used k-fold cross validation [57] on the augmented dataset to assess the generalization capabilities of our model and to observe whether there is any concern for overfitting. Overfitting occurs when a model has a relatively high training accuracy but much lower validation accuracy. A k fold cross validation is performed on the augmented dataset for ensuring that every observation from the original dataset has a chance of appearing in the training and test set. It is a standard practice to detect overfitting in a CNN model. We experimented with k values of 5 and 10 as these are conventional values [58] for k fold cross validation. Experimental results of 5-fold and 10-fold are shown in Table 15 and Table 16.

It is found that our proposed model achieved an average validation accuracy of 98.01% in 5-fold cross validation on an enhanced augmented mammography dataset. On the other hand, the model achieved an average validation accuracy of 97.90% using 10-fold cross validation. Most of the folds showed validation accuracies above 96% in both 5-fold and 10-fold cross validation. No fold displays an alarming difference between training and validation accuracies. Moreover, all training and validation accuracies are fairly balanced in every fold. This suggests that our method has the ability of yielding optimal performance without evidence of overfitting even in different shuffling scenarios.

### 8.6. Evaluation on Noise Induced Test Set

While dealing with real world mammograms, image quality is often found to be corrupted due to artefacts and noise. Since our proposed system performs with optimal accuracy on mammograms of a public dataset, it should be investigated how the model performs as noise increases. The presence of noisy images in a dataset can significantly affect the overall performance and tends to decrease classification accuracy. This phenomenon occurs due to the loss of important information in noisy data. The model is therefore tested on a dataset containing noisy mammograms. To create mammograms which are similar to real world noisy mammograms, we have added Gaussian noise [59] with a value of 0.1 [60] on preprocessed mammograms. Experimental result of noise induced dataset are shown in Table 17.

With the noise induced dataset, a slightly reduced accuracy of 95.87% and an F1 score of 95.44% are observed. While a reduction in accuracy is predictable, it is small demonstrating that our proposed approach can be expected to yield optimal performance even with real world noisy images.

### 8.7. Comparison with Previous Literature

In this section, we compare our model with some recent studies which were mentioned in the literature review section. Table 18 shows the best accuracy achieved by previous similar studies and our proposed model BreastNet18.

**Figure 24:**
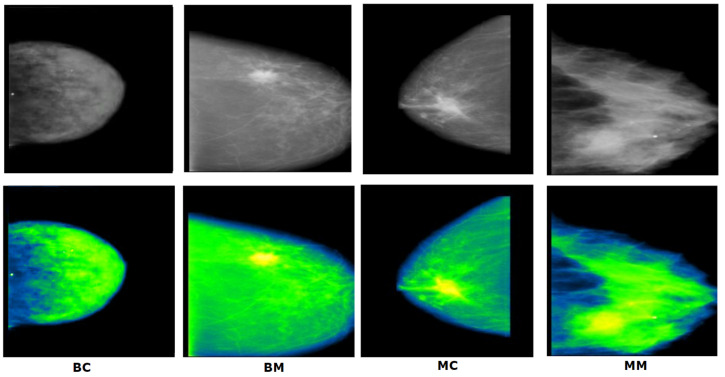
Examples of different classes based on nodules.

More than half of the studies shown in Table 18 used VGG16 and datasets similar to ours. Kamparia et al. [18], used transfer learning with their proposed MVGG model, based on the VGG16 image classification model, similar to our method. They used the DDSM database for mammograms. With their best model they were able to achieve an accuracy of 94.3%. Similar studies were described by others [17]. A fine-tuned VGG16 network with a FC layer resulted in an accuracy of 90.5% with optimizer Adam using the DDSM mammogram database. A fairly recent study conducted by Shallu and Mehra [25], used the DDSM database for training a fine-tuned pre trained VGG16. Their highest accuracy was 92.60%. Hameed et al. [22] described experiments with VGG16 and VGG19 achieving an accuracy of 94.71% with pre-trained VGG16. Li et al. [5] introduced some pre-trained models in their study and got a highest accuracy of 92.78% with VGGNet. All these studies achieved their highest accuracy by employing a VGG16 based model. However, the performance of two other CAD systems [23,28] was also compared with ours. For training purpose, they used CBIS-DDSM database and obtained an accuracy of 93.73% and 95.64% respectively, where [28] used optimizer Adam similar to ours. Furthermore, a comparative analysis of early detection of nodules, in previous literatures and our research has also been done. Wang et al. [61] developed a CAD system where a 3D U-Net model was used for breast nodules detection, and a multiple layer CNN for classification. Their proposed U-Net model achieved a sensitivity of 91%. The CNN model performed best achieving a classification accuracy of 87.5%, a sensitivity of 87.0% and a specificity of 88.0%. Matheus and Schiabel [62] designed a module to identify and categorize breast nodules on mammograms at an early stage. ROIs containing nodules were detected using a neural network. Segmentation of the detected nodule was carried out in order to classify benign and malignant cases. They achieved a detection sensitivity of 74% and a sensitivity of 81.28%. In breast imaging, low pixel resolution and the surrounding tissue, a small size of the nodule or a difficult location may all lead to poor performance [61]. In our research this issue is addressed by enhancing and highlighting the nodules in different pixel value and intensity as in Figure 24.

Figure 24 shows that our image processing techniques are able to successfully highlight potential cancerous nodules in different color intensity. This improved the effectiveness of our model. Our model outperforms the models described above with and accuracy of 98.02%, despite using a small dataset. By conducting image pre-processing and data augmentation, our proposed model is able to achieve an outcome that surpasses recent state of the art studies. This achievement demonstrates the potential of BreastNet18 in classifying breast cancer from mammograms with the highest accuracy.

## 9. Limitations

Our proposed approach is carried on a dataset containing a relatively small number of images. Though the number of images is increased by applying different data augmentation techniques; the performance of our proposed network could be further assessed using a larger dataset. Furthermore, in most cases, real world data differ from publicly available dataset. It could be explored how the model performs on real world data, if we could further experiment with real world data.

**Figure 25:**
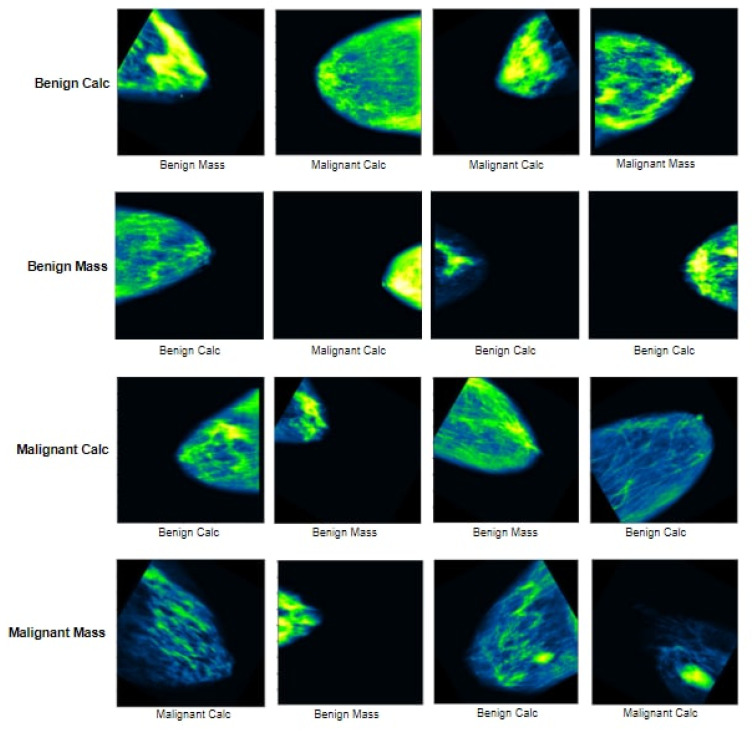
Example of misclassified images of all four classes.

The test dataset used in this research contains a total of 2306 mammograms (667 images in Benign calc, 636 in Benign Mass, 480 in Malignant Calc and 523 in malignant mass) and while testing our model with this dataset, it recorded a test accuracy of 98.02%. Fewer than 2% of the images are misclassified which is quite low compared to other studies mentioned in literature. From the confusion matrix it is observed that among 2306 mammograms, a total of 48 mammograms from all four classes are misclassified. Among them, the highest correct predictions are achieved in the Benign Mass (BM) class where only 5 images are misclassified. For the Malignant Mass (MM), a number of 11 images are misclassified and for Malignant Calc (MC), a total of 14 images are misclassified. The highest misclassification rate is noticed for Benign Calc with a total of 18 misclassified mammograms. Figure 25 illustrates an example of a misclassified image for all four classes.

One potential reason for these misclassifications can be that, even after applying multiple image processing techniques, the model still has some difficulties in distinguishing cancer cells from dense breast tissue. In Benign Mass, model misclassified a mammogram as Malignant Calcification due to the presence of dense tissue. Some of the images of Benign Mass have similarities with Benign Calcification. On the other hand, the model sometimes classifies Benign Calcification images as Benign Mass or Malignant Mass as some of the images of these two classes have a strong resemblance to the Benign Calcification images. The opposite effect can be observed with Malignant Calcification and Malignant Mass images. Some of the images of these two classes contain less dense tissue which the model misinterprets as Benign Calcification and Benign Mass. Another reason can be that there is a limited data diversity and in this number of images. Though, data augmentation increases the number of images, the model may not learn enough new features due to the lack of new data. However, in the majority of the test images the model performs very well by correctly classifying the cases. The model is robust despite of its minor limitations.

## 10. Contribution of This Study

The aim of this study is to develop a CNN model to classify breast cancer using CBIS-DDSM mammography dataset into four intra classes with highest possible accuracy. We have experimented with several deep learning models which are fine-tuned to carry out transfer learning in order to find the optimal network based on highest performance and lowest layers depth as well as less number of trainable parameters. The main challenges of this study include limited number of images, artefacts and noise present in mammograms and classifying cancer into four intra classes. Classifying cancer into benign and malignant is often easier to implement as the model is just predicting whether cancer is present or not. However, how a cancerous nodule forms from primary stage to critical stage is quite difficult to interpret as the similarity of lesion structure for intra classes is nearly close. Therefore, one of the main contributions of this study is the wide exploration of several standard image preprocessing techniques described in literature for mammogram classification. However, the effectiveness of an algorithm for a specific dataset greatly depends on image characteristics and the target application. Similar algorithm and technique may not provide similar and expected outcomes for different datasets, especially in mammography as the pattern and characteristics of mammography can be quite complex. Therefore, all the methods and their parameter values employed in this study are evaluated by conducting extensive experiments and ablation studies. Parameters values are chosen after thorough experimentation in order to maintain good PSNR scores and achieve optimal outcomes. We have showed how the changes of parameter values highly impacts on image output and why an optimal threshold value is crucial to determine and apply. Image processing algorithms namely binary masking, morphological opening, largest contour detection, inRange operation, Gabor filter, Gamma correction, CLAHE, Green fire blue filter are applied with the best suitable parameter values. To assess our techniques, a result analysis based on these image processing techniques is also added to evaluate how these methods greatly impact on overall performance. In this regard, one of our most effective approach is applying CLAHE twice and to the best of our knowledge, no study attempts to experiment in this way. We have explained that several parameter values are employed at the first place to get the desired outcome. However, necessary details were lost with altering the values. Afterwards, CLAHE is applied twice and it is found that in this way the cancerous lesion is properly enhanced. Another contribution regarding image processing technique is to highlight the cancerous lesion into different intensity level using Green Fire Blue filter. As mentioned, distinguishing nodules of different intra classes is challenging to carry out, this filter greatly impacts on highlighting the nodule, dense tissue and surrounding tissues into three different pixel intensity levels. Therefore, our approach is able to yield a good performance in early detection of nodule as well. To address the issue of limited number of images, several effective and suitable augmentation techniques for our dataset are used.

Apart from image processing and data augmentation techniques, this study also demonstrates how the performance of a DCNN can be boosted by ablation studies. Ablation studies include experimenting with various batch sizes, flatten layers, loss functions, optimizers, and learning rates. Our model outperforms the existing literatures in terms of accuracy which is demonstrated in literature review section. As several DCNN are employed, a well statistical analysis among the performances is shown and described. Though the loss and accuracy curve for the best performing model confirms absence of overfitting, for a rigorous check we have carried out k-fold cross validation technique with k values of 5 and 10. Results suggest that, against our high accuracy, the model has no occurrence of overfitting. However, as the proposed model performs with a high accuracy with no overfitting issues, it should be checked whether the approach can be appropriate in a real world dataset. As real world mammograms may have degraded quality and presence of noise due to technical fault, we have tested our model on noise induced test dataset and performance did not drop significantly. This simulation evidences that, in future real world mammography applications, our approach might be constructive and an improved performance might be attained following this approach. In summary, we have showed a complete and effective pathway of mammography classification from image preprocessing to attaining an optimal performance with the help of extensive experimentations and performance analysis. Furthermore, brief descriptions of all the techniques and ablation studies are given, which may help scholars to understand the challenges of mammography classification in similar dataset and effective ways to address them.

## 11. Conclusions

In this study, a breast CAD system is proposed to classify mammograms into four classes. Images used in this experiment contain noise and artefacts which were removed by using image pre-processing techniques. Background removal and image enhancement techniques were employed to further improve the quality of the raw mammograms. Six pre-trained and fine-tuned DCNN models were experimented with to determine which model performs with the highest accuracy. A BreastNet18 model is proposed employing VGG16 as foundational base, as VGG16 yields the highest accuracy. An ablation study is carried out on our proposed network, to evaluate and improve the model’s robustness. The pre-processed image dataset is augmented by seven augmentation techniques resulting in a dataset of 11536 mammograms. Our Proposed BreastNet18 model performed best with optimizer Adam and learning rate 0.0008, resulting in a training accuracy of 96.72%, a validation accuracy of 97.91% and a test accuracy of 98.02%. Our proposed approach based on image processing, transfer learning, fine-tuning and ablation study demonstrated the highest rate of correct classification while dealing with complex mammogram images. The findings of this study demonstrate that image processing and augmentation techniques can significantly increase a model’s performance. Transfer learning can be an appropriate approach in computer vision when working with a small number of images. In addition, configuring the model’s architecture and hyper-parameter employing an ablation study also has an impact on the overall accuracy. To sustain the robustness of model, it is tested on a noise induced dataset and overfitting is addressed by employing k fold cross validation. In both cases, an optimal performance is maintained. Our approach may aid clinical specialists in diagnosing and treatment planning at an early stage.

## Figures and Tables

**Table 1:** Dataset description.

Name	Description
Total Number of Images	1459
Dimension	224 × 224
Color Grading	RGB
Benign Mass	417
Benign Calcification	398
Malignant Calcification	300
Malignant Mass	344

**Table 2:** Selected parameter values for all pre-processing algorithms.

Process	Algorithm	Function/Method	Parameter Value
	Binary masking	cv2.rectangle()	Thickness = 5
	Morphological opening	cv2.threshold()	Threshold = 127, maximum = 255, type = cv2.THRESH_BINARY
		cv2.morphologyEx()	Type = cv2.MORPH_OPEN, kernel size = (20, 20)
Artefacts removal		cv2.threshold()	Threshold = 127, maximum = 255, type = cv2.THRESH_BINARY
	Largest contour detection	cv2.findContours()	Contour retrieval mode = cv2. RETR_EXTERNAL, contour approximation method = cv2.CHAIN_APPROX_SIMPLE
		max()	key = cv2.contourArea
		cv2.drawContours()	Index = −1, border color = (255, 255, 255), thickness = 1
	inRange Operation	cv2.inRange()	Lowest = (0, 0, 200), highest = (255, 255, 255)
	Gabor Filter	cv2.getGaborKernel()	Kernel size = (5, 5), sigma = 3, theta = 1*np.pi/1, lambd = 1*np. Pi/4, gamma = 0.5, phi = 20 and ktype=cv2.CV_32F
Remove line		cv2.filter2D()	Source image, kernel
	Morphological opening	cv2.getStructuringElement()	Structuring element = cv2.MORPH_RECT, kernelSize = (1, 30)
		cv2.morphologyEx()	morphological operation = cv2.MORPH_OPEN
	Morphological dilation	cv2.dilate()	kernelSize = (5, 5)
Image enhancement	Gamma correction	np.array()	Gamma value = 2.0
	CLAHE	cv2.createCLAHE()	clipLimit =1.0, tileGridSize = (8, 8)

**Table 3:** MSE, PSNR, SSIM, and RMSE values for ten images.

Image	MSE	PSNR	SSIM	RMSE
Image_1	15.37	37.67	0.962	0.13
Image_2	15.63	37.29	0.961	0.13
Image_3	14.25	38.28	0.964	0.12
Image_4	13.31	37.31	0.962	0.13
Image_5	13.37	39.67	0.967	0.11
Image_6	11.53	41.29	0.973	0.09
Image_7	13.63	39.28	0.963	0.11
Image_8	12.47	40.36	0.969	0.10
Image_9	15.21	37.79	0.963	0.13
Image_10	13.82	39.31	0.966	0.11

**Table 4:** Performance comparison of transfer learning models on preprocessed augmented mammogram dataset.

Classifier Name	Pre (%)	Recall (%)	F1 (%)	Spe (%)	Sen (%)	Tr_Acc (%)	Tr_Loss (%)	Val_Acc (%)	Val_Loss (%)	Ts_Acc (%)	Ts_Loss (%)
MobileNetV2	77.93	77.78	77.85	78.15	77.91	78.72	0.327	77.62	0.375	77.84	0.366
ResNet50	80.12	79.91	80.01	81.21	81.35	80.43	0.380	79.81	0.324	79.98	0.301
DenseNet201	87.06	86.83	86.94	88.21	88.63	88.03	0.292	86.77	0.284	86.92	0.271
InceptionV3	76.74	76.84	76.78	78.85	79.91	76.91	0.421	76.21	0.392	76.87	0.384
VGG16	97.29	97.02	97.15	98.03	98.14	97.79	0.182	96.83	0.115	97.13	0.081
VGGNet19	96.33	96.27	96.29	97.33	97.17	96.63	0.221	95.63	0.205	96.24	0.197

**Table 5:** Statistical result analysis of the models.

Classifier Name	FPR (%)	FNR (%)	FDR (%)	KC (%)	MCC (%)
MobileNetV2	21.15	22.22	22.07	78.65	68.56
ResNet50	18.79	20.09	19.88	80.63	70.48
DenseNet201	11.79	13.17	12.94	86.27	77.25
InceptionV3	21.85	23.16	23.26	75.81	67.02
VGG16	1.97	2.98	2.71	98.52	88.62
VGGNet19	2.67	3.73	3.67	97.46	86.50

**Table 6:** Result analysis of the models based on error matrices.

Classifier Name	MAE	RMSE
MobileNetV2	17.09	32.57
ResNet50	15.37	36.51
DenseNet201	11.78	26.65
InceptionV3	19.32	44.36
VGG16	2.44	7.20
VGGNet19	3.16	12.51

**Table 7:** Specifications of the models.

Model	Layer Depth	Trainable Parameters
VGG19	19	50,178
Mobilenetv2	53	125,442
ResNet50	50	200,706
InceptionV3	48	102,402
DenseNet201	201	188,162
VGG16	16	50,178

**Table 8:** Ablation study by changing Flatten layer.

Case Study	Layer Name	Val_Acc (%)	Val_Loss (%)	Ts_Acc (%)	Ts_Loss (%)	AUC (%)	Finding
	Flatten	96.98	0.12	97.13	0.12	97.57	Identical performance
1	GlobalAveragePooling2D	96.98	0.12	97.13	0.12	97.57	Identical performance
	GlobalMaxPooling2D	96.02	0.19	96.56	0.19	97.34	Accuracy dropped

**Table 9:** Ablation study by changing Batch size.

Case Study	Batch Size	Val_Acc (%)	Val_Loss (%)	Ts_Acc (%)	Ts_Loss (%)	AUC (%)	Finding
	16	96.98	0.16	97.44	0.197	97.72	Accuracy increased
2	32	97.03	0.09	97.57	0.064	97.98	Accuracy increased
	64	96.83	0.11	97.13	0.081	97.57	Identical accuracy

**Table 10:** Ablation study by changing Loss function.

Case Study	Loss Function	Val_Acc (%)	Val_Loss (%)	Ts_Acc (%)	Ts_Loss (%)	AUC (%)	Finding
	Categorical Crossentropy	97.03	0.09	97.57	0.064	97.98	Identical accuracy
3	Cosine similarity	96.86	0.12	96.93	0.05	96.94	Identical accuracy
	Mean Squared Error	95.64	0.18	96.72	0.08	96.86	Accuracy dropped

**Table 11:** Ablation study by changing Optimizer and learning rate.

Case Study	OP	LR	Val_Loss (%)	Val_Acc (%)	Ts_Loss (%)	Ts_Acc (%)	AUC (%)	Findings
	Adam	0.006	0.56	94.89	0.3110	95.0997	95.63	Accuracy dropped
		0.001	0.09	97.03	0.06	97.57	97.98	Identical accuracy
4		0.0008	0.14	97.03	0.05	98.02	98.27	Accuracy increased
	Nadam	0.006	0.42	95.15	0.26	96.18	96.94	Accuracy dropped
		0.001	0.14	96.62	0.08	97.31	97.67	Accuracy dropped
		0.0008	0.14	96.19	0.08	97.48	98.15	Accuracy dropped
	Adamax	0.006	0.18	95.50	0.10	96.61	96.82	Accuracy dropped
		0.001	0.18	94.89	0.14	95.35	95.46	Accuracy dropped
		0.0008	0.19	94.29	0.15	94.96	95.27	Accuracy dropped
	RMSprop	0.006	0.83	93.94	0.44	95.35	95.53	Accuracy dropped
		0.001	0.20	94.46	0.14	94.27	94.65	Accuracy dropped
		0.0008	0.22	94.24	0.12	94.10	95.68	Accuracy dropped

**Table 12:** Performance analysis for different imageJ filters.

Filter	Val_Acc (%)	Test_Acc (%)	F1_score (%)
Green fire blue	97.03	98.02	98.15
Blue orange icb	94.78	95.23	95.27
6 shades	94.42	94.11	94.25
16 colors	95.43	95.87	95.93

**Table 13:** Result of ablation study based on image preprocessing algorithms.

Experiment	Test Accuracy (%)	Validation Accuracy (%)	F1_Score (%)
Raw image	79.32	72.86	79.36
Artefact removal	88.45	87.38	88.62
CLAHE 1ST	94.63	91.67	94.74
CLAHE 2ND	95.21	93.07	93.28
Green fire blue	98.02.	97.03	98.15

**Table 14:** Configuration of proposed architecture after ablation study.

Configuration	Value
Image size	224 × 224
Epochs	90
Optimization function	Adam
Learning rate	0.001
Batch size	32
Weight decay	0.0001
Activation function	Softmax
Dropout	0.5
Momentum	0.9

**Table 15:** Result of 5-Fold cross validation.

Accuracy	Fold 1	Fold 2	Fold 3	Fold 4	Fold 5	Average Validation Accuracy
Training	97.93	99.35	98.83	98.57	99.06	
Validation	96.85	98.71	97.74	98.26	98.53	98.01

**Table 16:** Result of 10-Fold cross validation.

Accuracy	Fold 1	Fold 2	Fold 3	Fold 4	Fold 5	Fold 6	Fold 7	Fold 8	Fold 9	Fold 10	Average Validation Accuracy
Training	97.34	99.06	97.40	99.15	98.83	98.72	98.86	99.07	98.47	98.60	
Validation	97.48	98.63	96.81	98.72	97.95	97.64	98.54	98.26	96.88	98.15	97.90

**Table 17:** Experimental result on noisy dataset.

Noise	Amount	Optimizer	Learning Rate	Test Accuracy (%)	F1_Score (%)
Gaussian	0.1	Adam	0.001	95.87	95.44

**Table 18:** Accuracy comparison with existing literature.

Work	Model	Dataset	Batch	Epoch	Optimizer	Learning Rate	Accuracy
Nasir Khan et al. (2019) [23]	MVFF CADx	CBIS-DDSM: 3568 mammograms & MIAS: 322 mammograms	32	100	SGD	0.001	93.73%
Al-antari et al. (2018) [28]	Fully Integrated CAD	INbreast Toward a Full-field Digital Mammographic Database: 410 mammograms After augmentation: 896	24	100	Adam	0.0001	95.64%
Hameed et al. (2020) [22]	Full trained VGG16 + VGG19 Fine-tuned VGG16 + VGG19	Pathology department of Colsanitas Colombia University provided 544 image of breast cancer.	32	200	Adam	0.0001	93.53% 95.29%
Khamparia et al. (2021) [18]	Hybrid MVGG16 ImageNet	Digital Database for Screening Mammography (DDSM) containing 2620 mammograms.	32	15	-	-	94.3%
Institute of Electrical and Electronics Engineers, n.d. [17]	Pre-trained VGG16 with 1 FC layer	Digital Database for Screening Mammography (DDSM) containing 2620 mammograms. Mammographic Image Analysis Society containing 332 mammograms.	20 15	500 500	Nadam RMSprop	-	90.5% 91.2%
Shallu and Mehra, (2018) [25]	VGG16 + LR VGG19 + LR ResNet50 + LR	BreakHis dataset used in collaboration with the Prognostic and Diagnostic Laboratory containing 7909 breast cancer images.	-	-	-	-	92.60% 90.40% 79.40%
Li et al. (2019) [5]	AlexNet VGGNet GoogLeNet DenseNet DenseNet-II	The First Hospital of Shanxi Medical University provided 2042 full-field digital mammograms. With augmentation the data set was increased to 30,630 images	-	-	-	0.01	92.70% 92.78% 93.54% 93.87% 94.55%
Our work	BreastNet18	Pre trained on ImageNet dataset consisting of over 14 million images. CBIS-DDSM dataset: 1442 mammograms. After augmentation: 11,536 mammograms.	32	350	Adam	0.0008	98.02%

## Data Availability

The Cancer Imaging Archive (TCIA) dataset [35] is publicly available.
